# Prediction accuracy and repeatability of UAV based biomass estimation in wheat variety trials as affected by variable type, modelling strategy and sampling location

**DOI:** 10.1186/s13007-024-01236-w

**Published:** 2024-08-20

**Authors:** Daniel T. L. Smith, Qiaomin Chen, Sean Reynolds Massey-Reed, Andries B. Potgieter, Scott C. Chapman

**Affiliations:** 1 School of Agriculture and Food Sustainability, The University of Queensland, St Lucia, QLD 4072 Australia; 2 Center for Crop Science, Queensland Alliance for Agriculture and Food Innovation, The University of Queensland, St Lucia, QLD 4072 Australia

**Keywords:** Wheat biomass estimation, UAV based monitoring, High throughput phenotyping, Field-based

## Abstract

**Background:**

This study explores the use of Unmanned Aerial Vehicles (UAVs) for estimating wheat biomass, focusing on the impact of phenotyping and analytical protocols in the context of late-stage variety selection programs. It emphasizes the importance of variable selection, model specificity, and sampling location within the experimental plot in predicting biomass, aiming to refine UAV-based estimation techniques for enhanced selection accuracy and throughput in variety testing programs.

**Results:**

The research uncovered that integrating geometric and spectral traits led to an increase in prediction accuracy, whilst a recursive feature elimination (RFE) based variable selection workflowled to slight reductions in accuracy with the benefit of increased interpretability. Models, tailored to specific experiments were more accurate than those modelling all experiments together, while models trained for broad-growth stages did not significantly increase accuracy. The comparison between a permanent and a precise region of interest (ROI) within the plot showed negligible differences in biomass prediction accuracy, indicating the robustness of the approach across different sampling locations within the plot. Significant differences in the within-season repeatability (w^2^) of biomass predictions across different experiments highlighted the need for further investigation into the optimal timing of measurement for prediction.

**Conclusions:**

The study highlights the promising potential of UAV technology in biomass prediction for wheat at a small plot scale. It suggests that the accuracy of biomass predictions can be significantly improved through optimizing analytical and modelling protocols (i.e., variable selection, algorithm selection, stage-specific model development). Future work should focus on exploring the applicability of these findings under a wider variety of conditions and from a more diverse set of genotypes.

**Supplementary Information:**

The online version contains supplementary material available at 10.1186/s13007-024-01236-w.

## Introduction

Utilizing secondary physiological traits in grain crops to adapt to the environment is a crucial approach for securing food supply in an increasingly unpredictable world [52]. Biomass formed during the vegetative stage of crop development acts as a potential source of resources that can be translocated into harvestable yield [53]. Under non-limiting conditions, there is a positive relationship between biomass accumulation and grain yield [46], whereas excessive biomass formation under water or nutrient limitation may lead to asynchrony between resource use and availability, with potential to impede final yield. In wheat, biomass, along with fraction of intercepted radiation, is a key variable in defining radiation use efficiency (RUE), which relates to the ability of a plant to capture, and harness photosynthetic energy to produce the photosynthates necessary for growth [62]. The accurate monitoring of in-season biomass dynamics can thus provide a deeper understanding of the physiological dynamics of crop performance, especially when examined through the lens of genetic, environmental, and management-based interactions. This can lead to more appropriate selection of varieties more suitable for the target population of environments (TPE) for which they are destined [8].

Physical assessment of biomass formation at a plot level is laborious, time consuming and limited by the need for destructive sampling in the field [17]. The logistical difficulties and time constraints associated with measuring large numbers of biomass samples, mean that they are typically taken at a few key stages of crop growth, and from a limited number of plots. These limitations make it difficult to evaluate the differences between genotypes in large trials and where multiple environments are involved. As a result, approaches to estimating biomass using high throughput phenotyping (HTP) have become commonplace for a wide range of species, for example, in wheat [14, 16, 32], sorghum [36], maize [79] and soybean [75]. A large body of research has been conducted to develop indirect methods for the prediction of biomass using effective information extracted from RGB [39], multispectral [38], hyperspectral [75] and LIDAR sensors [13, 14]. Indirect methods for biomass prediction provide opportunity for repeatable measurements that are less prone to human error, and are scalable, making them more amenable to use in large-scale variety selection programs [64].

Biomass prediction has been approached by modelling univariate relationships with HTP features, or more complex multivariate approaches. For example, [4] used UAV-based crop height as input for biomass prediction in the simple linear model. Such a univariate regression model presents an explicit biological relationship but cannot take advantage of the complimentary nature of data obtained from multiple sources [18]. Data fusion has been shown in many studies to improve the prediction accuracy for crop traits [47, 50, 76]. However, a major consideration in such datasets is they are often high dimensional and include both redundant and irrelevant variables, which reduce model interpretability and performance whilst increasing training time [26, 35]. Thus, effective variable selection is vital in optimizing model development to improve model performance for predicting target crop traits (e.g., biomass) using high-dimensional HTP datasets.

In addition to variable selection, several factors regarding model selection have implications for biomass prediction accuracy. First, the algorithm used to develop prediction models play a key role, and machine learning models have been demonstrated to improve the accuracy of predictions over traditional statistical methods for a wide range of regression-related tasks [63]. Second, a major challenge in predictive modelling surrounds the trade-off between model specificity and generalizability. While a model that is trained on as many data points as possible, using a wide variety of growth stages and geographic locations, might be widely generalizable (scalability), the ability of such a model to identify important nuances in the data may be reduced. As such, multiple strategies exist for modelling experiment- level biomass: A single model could be trained across all available experiments and time-points with a focus on generalizability, or multiple models could be trained for individual circumstances (i.e., different growing stages, management practices, or years), with the aim of enhancing specificity. There is also the practical question of how to obtain high precision in the field, perhaps by establishing a ‘global’ prediction model, and enhancing it by a small set of biomass sampling in any given experiment, i.e., ‘real-time calibration’ by using a subset of the plots to build an enhanced model or check a ‘global’ model [29].

Another standing question in field-based phenotyping surrounds the region of interest (ROI) that image features are analysed [61]. While there are a range of options for automating the creation of ROIs within HTP workflows [70], the impact of the location of the ROI within the plot has not received sufficient attention. While biomass itself is only typically taken from a small area within a plot, provided the plot is homogeneous, it is often assumed that the ROI should be representative enough to correlate with the location where the sample was taken [28, 59]. This approach has the advantage of allowing repeated measures over time, which may not be a major issue where within-plot variability is low, however, this may become an issue that reduces model accuracy where plot variability is high, for example [59]. Thus, how well a permanent ROI represents ground-truth measurements, and whether other approaches, such as sampling UAV indices directly from where the trait was measured would be more appropriate, remains an important factor to consider in HTP workflows.

In the context of variety selection, the goal of any phenotypic assessment is to determine genotypic differences to help understand the physiological basis of performance (i.e., yield) [17, 52]. While in the earlier stages of a breeding program, genotypic differences in performance can be wide, given the diversity in early populations, at later stages, the variation can be reduced as selection may favour similar physiological adatptation pathways. While many studies have explored biomass prediction where variability is high, either at earlier stages of a breeding program [71], or where experimental treatments have been imposed to increase variability [4], we are not aware of studies that have attempted to estimate biomass between commercial varieties where variability is lower.

Given the spatial variability inherent in the field, there are a range of factors that can confound genetic effects, such as soils, moisture, shade, slope, and management [27]. To account for this variability, experimental design [9] and post-hoc analyses using linear mixed models is often a necessary step [56]. Based on this process, the genetic variance (V_G_) and residual variance (V_R_) of a trait can be estimated and used to predict within-season repeatability (w^2^). While many studies have used w^2^ to evaluate the efficacy of a prediction model [66], how these factors vary with regards to a model’s prediction accuracy has to our knowledge, not been explored [60].

In light of the issues surrounding variable selection, ROI determination and the estimation of w^2^ for HTP derived predictions, in this paper we explored the sensitivity of biomass prediction models in wheat to a range of factors, by (a) exploring the effects of variable selection and various popular analytical algorithms to build prediction models (b) investigating the impact of within-plot position used to derive predictive variables; (c) making comparisons between a generic model and stage/experiment specific models to better understand the trade-offs between generalisability and specificity, and (d) Computed w^2^, V_G_ and V_R_ for each growth stage and experiment to better understand the optimal timing of measurements. 

## Methods

### Field experiment

**Fig. 1:**
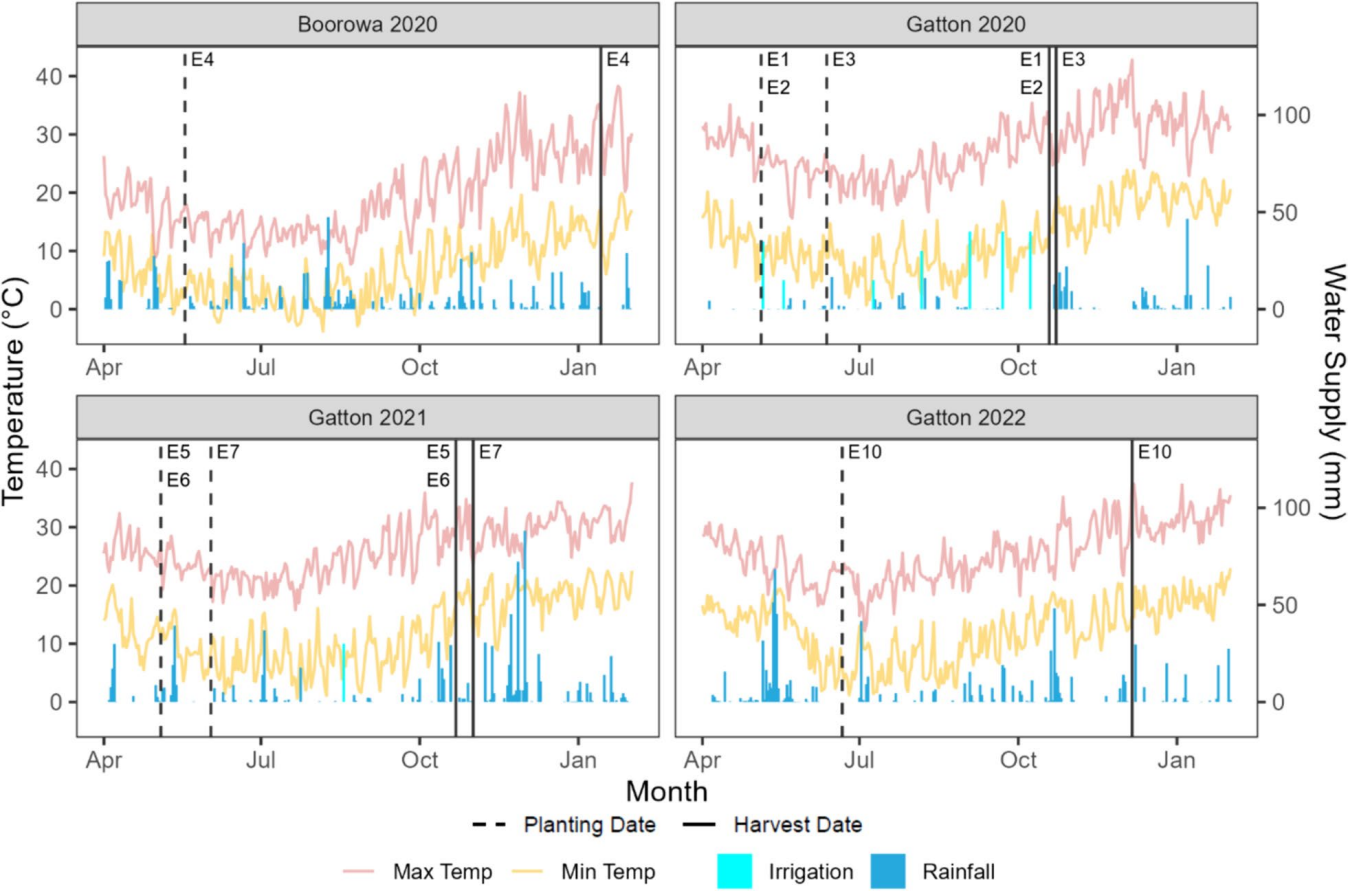
Minimum temperature (Min Temp) and maximum temperature (Max Temp), rainfall, and irrigation for each location and year combination. Dashed vertical lines represent planting dates and solid vertical lines represent harvest dates for individual experiments for a particular site and year combination. E1-E3 represent Gatton experiments in 2020, E4 represents Boorowa in 2020, E5-E7 represent Gatton experiments in 2021, E10 represents Gatton experiment in 2022

Wheat experiments were grown at Gatton Research Station, Queensland (27.56°S, 152.33°E) in 2020─2022 and at Boorowa Research Station, NSW (34.47°S, 148.69°E) in 2020. These sites contrast in the timing of rainfall and their temperature regimes throughout the season. Gatton receives most rainfall in the summer months leading up to the wheat growing season, and available crop water is normally dependent upon water stored in the soil profile during a summer fallow period. Alternatively, Boorowa typically receives rainfall during the growing season (Fig. 1). Gatton experiences higher in-season maximum and minimum temperatures than Boorowa, resulting in more rapid phenological development and a shorter growing season spanning May to October, whereas Boorowa, with its lower latitudes, experiences cooler mean temperatures that result in a longer growing season. While Gatton has a heavy, dar k Vertosol, Boorowa has a sandy loam with a sandy clay-loam subsoil described as a chromosol [42].

Table 1 describes the field experiments that were used for this study. At Gatton research station, three experiments (i.e., Early sowing date with high nitrogen, early sowing date with standard nitrogen, and a standard sowing date with standard nitrogen) were planted in 2020 and 2021, while a single experiment (i.e., late sown standard nitrogen) was planted in 2022. At Boorowa research station, one experiment (i.e., standard sowing standard nitrogen) was planted in 2020. Whereas Boorowa was grown under rainfed conditions, the Gatton site received supplementary irrigation to avoid the effects of drought stress. For each experiment, a variety trial (NVT) was planted, containing pre-commercial and commercial spring wheat varieties deemed suitable for the local environment by the GRDC NVT program (https://nvt.grdc.com.au). Compared to the original progeny of parental crosses and the several stages of selection that have been made by commercial breeders, these genotypes might be termed ‘elite’ given their history of selection.

**Table 1:** Agronomic characteristics of the 8 trials used in this study

Experiment	Location	Year	Sowing date	Harvest date	N fertilization (kg/ha)^a^	Total water supply (mm)^b^	NVT genotype number
E1	Gatton	2020	5 May (early)	19 Oct	300 (high)	319	30
E2	Gatton	2020	5 May (early)	19 Oct	100 (standard)	319	30
E3	Gatton	2020	12 June (standard)	23 Oct	300 (high)	271	18
E4	Boorowa	2020	18 May (standard)	14 Jan	100 (standard)	596	39
E5	Gatton	2021	4 May (early)	22 Oct	300 (high)	343	36
E6	Gatton	2021	4 May (early)	22 Oct	100 (standard)	343	36
E7	Gatton	2021	2 June (standard)	01 Nov	300 (high)	274	30
E10	Gatton	2022	21 June (late)	06 Dec	100 (standard)	378	36

^a^Applied at time of sowing – Urea N equivalent

^b^Cumulative in-season rainfall and irrigation

The NVTs were designed with a row column configuration with 3 replicates using the R package DiGGer [9]. Additionally, a biomass calibration trial (BioCal) accompanied each NVT. In 2020 and 2021 for Gatton (E1, E2, E3, E5, E6 and E7), BioCal trials consisted of 6 genotypes × 3 densities (75, 150 and 225 plants per metre), in addition to a ‘check’ genotype planted again at 150 plants per meter, and 187 and 112 plants per meter. This resulted in a total of 21 plots for the BioCal trials. In 2022 (E10), the Gatton BioCal trial consisted of 8 genotypes × 3 densities (75, 150 and 300 plants per meter), with 7 out of 8 genotypes replicated once, and a single ‘check’ variety replicated twice (resulting in a total of 27 plots).

### Biophysical measurements

For each experiment, dry weight of aboveground biomass (*DW*_*AGB*_) was measured by cutting plants at ground level within a quadrat area (*Quad*_*area*_) of a known size (Since row spacing and number varied, the quadrat area from which the sample was made varied for each experiment. The fresh weight of each sample was measured (*FW*_*quad*_) and the fresh weight of a subsample consisting of approximately 20 stems was taken (*FW*_*sub*_). This sample was oven dried at 750C until reaching a constant weight (DW_sub_). DWAGB was thus calculated as:

1 $$DW_{AGB} = { }\frac{{FW_{quad} {*}\left( {\frac{{DW_{sub} }}{{FW_{sub} }}} \right)}}{{Quad_{area} }}$$

**Fig. 2:**
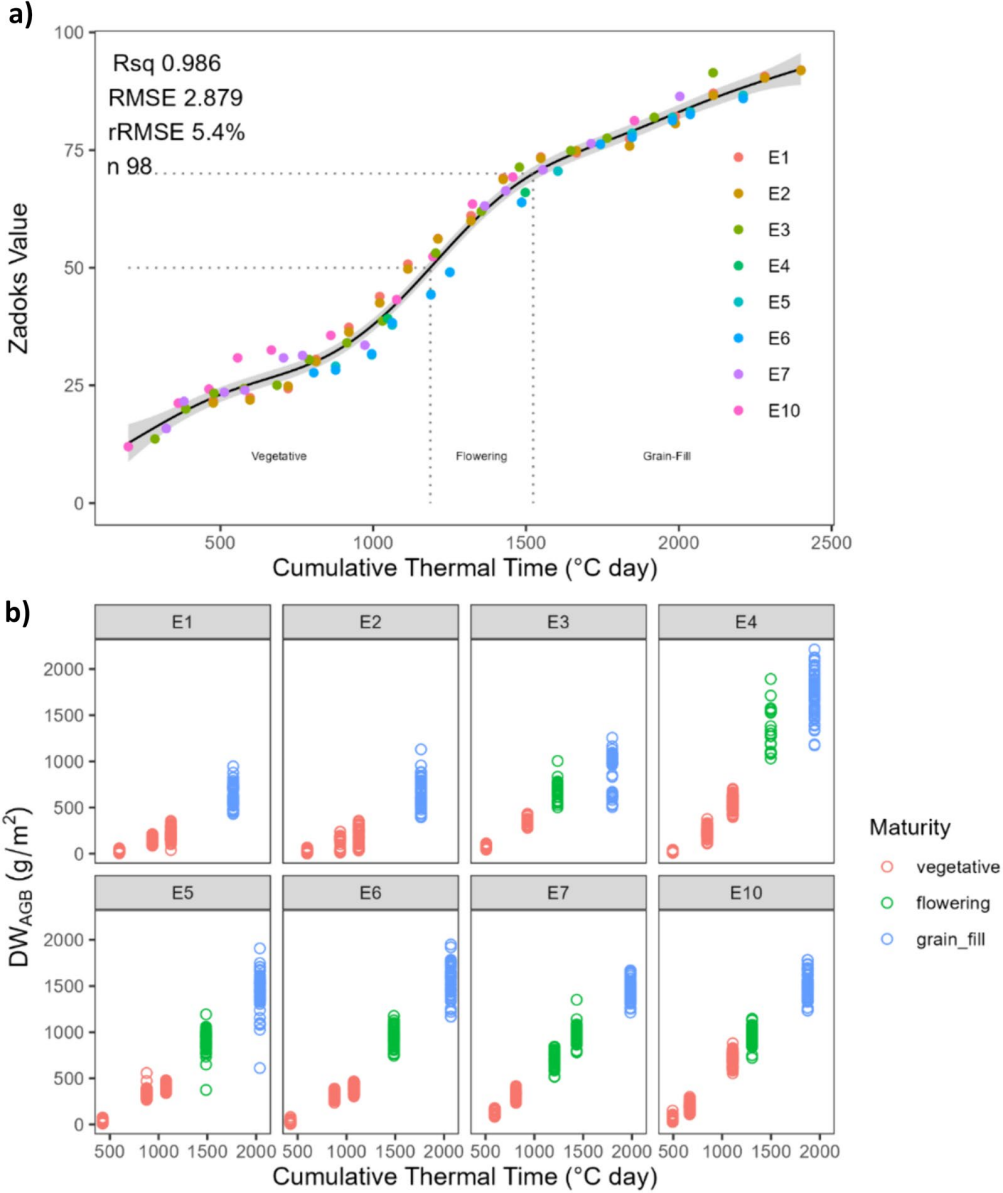
**a** Relationship between trial mean Zadok’s value and Cumulative thermal time (°C day) for measurements taken in each experiment (see coloured points). The solid line represents the line of best fit for a generalized additive model (GAM) and **b** the relationship between Cumulative thermal time (°C day) and aboveground biomass (DW_AGB_) for each experiment in the study, with point colours representing the Growth-stage as classified

At the time of each cut, biomass was measured from every plot within the BioCal trial, and additional biomass cuts were taken from a single replication of the NVT. In Gatton, growth stage was measured from only BioCal plots in 2020, and both BioCal and NVT plots in 2021 and 2022, using the Zadok’s growth scale on a weekly basis [77]. In Boorowa, Zadok’s growth scale was determined on two separate dates. To assign a particular DWAGB sample with a growth stage, the trial mean Zadok’s stage was calculated on each date with a Zadok’s score. Then, a generalized additive model (GAM) was built with using the following notation.

2 $$Y = { }\beta_{0} + f\left( {TT_{cumulative} } \right) + { } \in$$

where $Y$ represents the dependent Variable (Zadok’s stage), ${\beta }_{0}$ represents the intercept term, and $f({TT}_{cumulative})$ represents the smoothing function of the predictor variable Cumulative thermal time (${TT}_{cumulative}$) using a spline made up of third-degree polynomial segments joined smoothly using 10 knots. TT_cumulative_ and was calculated as per the method outlined by [80]. Using the relationship between TT_cumulative_ and predicted Zadok’s stage (illustrated in Fig. 2), growth stage was determined to be ‘vegetative’ where Zadok’s was between 11 and 49 (1-leaf stage up to the end of booting), ‘flowering’ was determined where Zadok’s values were between 50 and 69 (head emergence to the end of anthesis), and ‘grain-fill’ where Zadok’s values fell between 70 and 99 (milk stage to the end of ripening). Solving for the equation of the GAM model resulted in categorizing the crop stages as follows: Vegetative stage when TT_cumulative_ was less than or equal to 1189 °C day, Flowering stage when TT_cumulative_ was greater than 1189 °C day but less than or equal to 1523 °C day, and Grain-Fill stage when TT_cumulative_ exceeded 1523 °C day.

### Image collection and processing

Unmanned Aerial Vehicle (UAV) flights were performed as close in time as logistically possible to the biomass cuts. In E4 (Boorowa), the mean difference between biomass cuts and flight dates was 4.75 days due to the remoteness of the site, whereas the mean difference for Gatton experiments was 1.15 days, with the final cut during grain-fill for E3 an outlier with 7 days difference (see Fig. S1 for overview of flight dates and biomass cut dates). Fixed ground control points were placed within each experiment and their GPS coordinates were measured using Propeller Aeropoints (Propeller, Australia). All flights were performed within 2 h of solar noon, under full sunlight and with a wind speed of less than 10 km/hr. (see Table 2 for an overview of flight parameters). In Gatton 2021 and 2022, a DJI M300 was used, which allows for the use of both an RGB and Multispectral sensor simultaneously in the same flight. In Gatton and Boorowa in 2020, two separate flights had to be made, given the fact that the RGB and multispectral sensors were carried on separate UAVs. For RGB flights, shutter speeds of ≤ 1/1600th of a second were used to reduce motion blur, and a front and side overlap of 80% was used for all flights to ensure sufficient matching of pixels between adjacent images. All multispectral imagery was radiometrically calibrated using a nadir image of a MicaSense calibration panel taken before and after each flight. A total of 36 RGB and 36 Multispectral datasets were processed for this study.

**Table 2:** Overview of flight heights, multispectral and RGB sensor types and ground sample distances (GSD)

Experiment	Flight height (m)	Multispectral sensor	Multispectral GSD (cm)	RGB sensor	RGB GSD (cm)
E1, E2, E3	25	DJI P4M	1.33	DJI P4P	0.69
E4	25	MicaSense RedEdge MX	1.04	DJI P4P	0.69
E5, E6, E7, E10	20	MicaSense Altum	0.86	DJI Zenmuse P1 35 mm	0.25

**Fig. 3:**
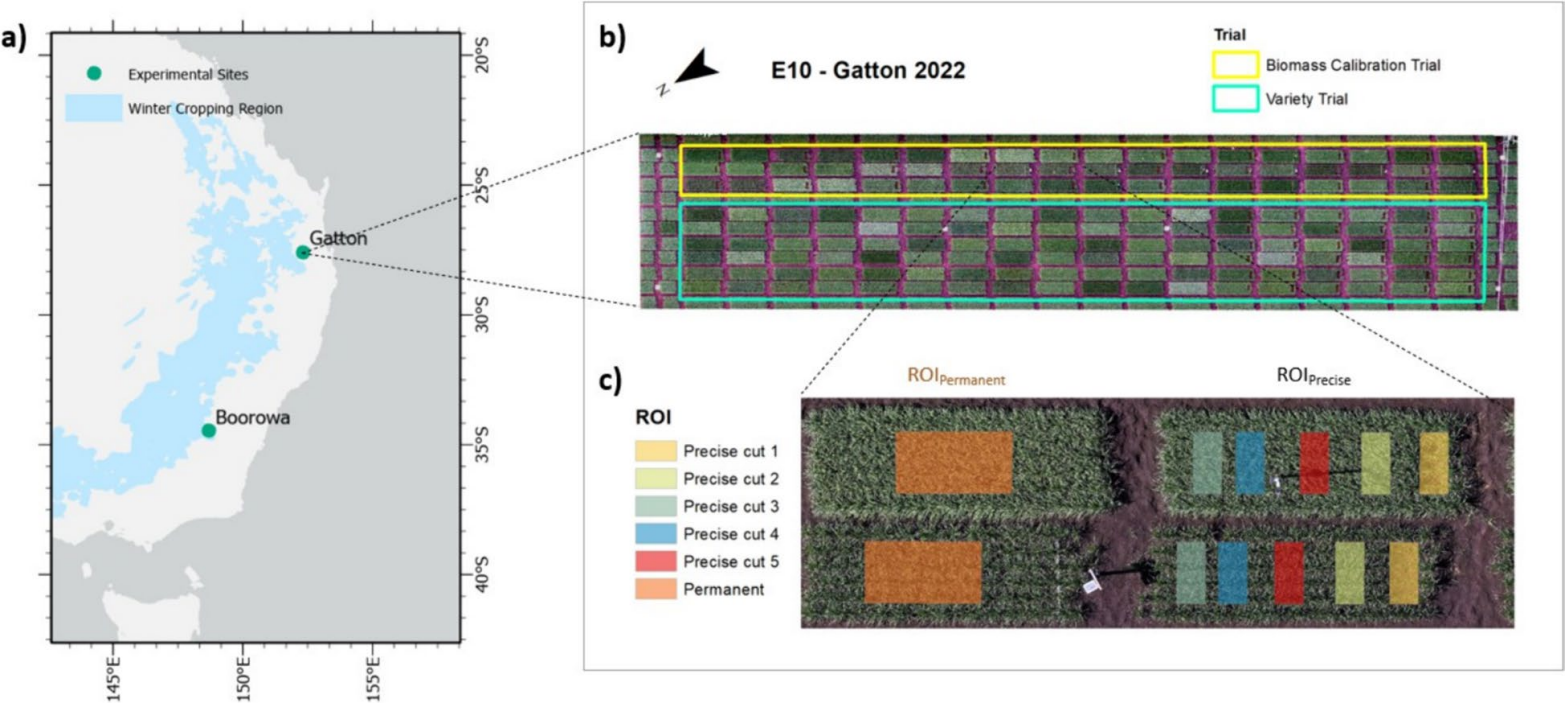
**a** Locations of Gatton and Boorowa sites in relation to the eastern and southern winter cropping region of Australia, **b** Gatton 2022 trial (E10), which includes a Biomass Calibration and NVT and **c** an example of paired plots with both an ROI_permanent_ which was repeatedly measured for each biomass cut across the season and five ROI_precise_ which were each analysed once for their respective biomass cuts

Raw imagery from each mission was processed using Agisoft Metashape (Agisoft, St Petersburg, Russia) which uses a structure from motion (SfM) algorithm to produce a 3d reconstruction of a scene from a set of images. First, multispectral imagery was calibrated through Metashape’s ‘calibrate reflectance’ option, which involves interpolation of the relationship between the known reflectance values of calibration panels and the timestamp of the before and after calibration panel images. This allows for in-flight reflectance values to be corrected based on estimated reflectance at a given time-point. Subsequently, Metashape was used to produce point clouds, which allow georectification and subsequent orthomosaic and digital elevation model (DEM) generation. A high degree of accuracy is enabled through SfM processing due to the presence of GCPs in the field. The average marker error across RGB orthomosaics was 1.62 cm and for MS orthomosaics was 1.37 cm. For each biomass cut taken within a plot, two ROIs were created for analysis: an ROI directly above the area where the biomass cut was taken (ROI_precise_), and an ROI in a section of the plot that was not to be disturbed at any time (ROI_permanent_) throughout the experiment until final harvest (see Fig. 3 for example trial design and illustration of the different ROIs used for the study). Each ROI_precise_ was created by manually locating the location of biomass cut using the closest orthomosaic generated after the biomass sampling. This process was completed using ArcMap.

### Variable extraction from proximal sensing

For both ROI types (ROI_precise_ and ROI_permanent_), spectral traits and geometric traits were calculated using a Python pipeline described by [12]. The spectral traits included 66 (33 × 2) variables, consisting of the median value for 33 vegetation indexes (Spectral traits) calculated from the entire or masked area within the ROI of the multispectral orthomosaic images. For the formulas of these Spectral traits refer to Table S4. The masked area represents the fraction of green plant matter within the ROI, which was distinguished based on a specific threshold value (OSAVI_threshold_) of optimized soil-adjusted vegetation index (OSAVI) (i.e., OSAVI > OSAVI_threshold_ for green plant matter, while OSAVI < OSAVI_threshold_ for soil background). The threshold value was calculated by using Otsu’s method [49] and OSAVI was selected for this purpose due to its demonstrated ability to distinguish between green plant matter and soil across phenological stages [43]. For example cases of the thresholding results images please refer to figure Fig. S2.

For each ROI type, the geometric traits (9) included 4 height-related variables, 3 area-related variables, 1 volume-related variable, and 1 coverage-related variables (refer to Table S5 for details). The height-related variables consisted of the 50th, 75th, 95th and 98th percentile values as well as standard deviation of canopy height across the entire ROI. The canopy height was calculated by subtracting pixel values of the crop surface model (CSM) by corresponding pixel values of digital terrain models (DTM). A date specific CSM was derived from the 3d point cloud created using RGB images; while the DTM was produced from a fight made at the beginning of the season (before emergence). The area-related variables consisted of the area within the ROI where the height values were below the 25th, 50th, 75th percentiles. The volume-related variable indicated the sum of the pixel heights within a given ROI multiplied by the ground sample distance, divided by the area of the ROI. The coverage-related variable indicated the proportion of masked area of the total area within ROI, where the masked area was distinguished based on the Otsu threshold values of OSAVI (see Table S5 and Fig. S2 for further details).

### Feature selection and biomass prediction models

**Fig. 4:**
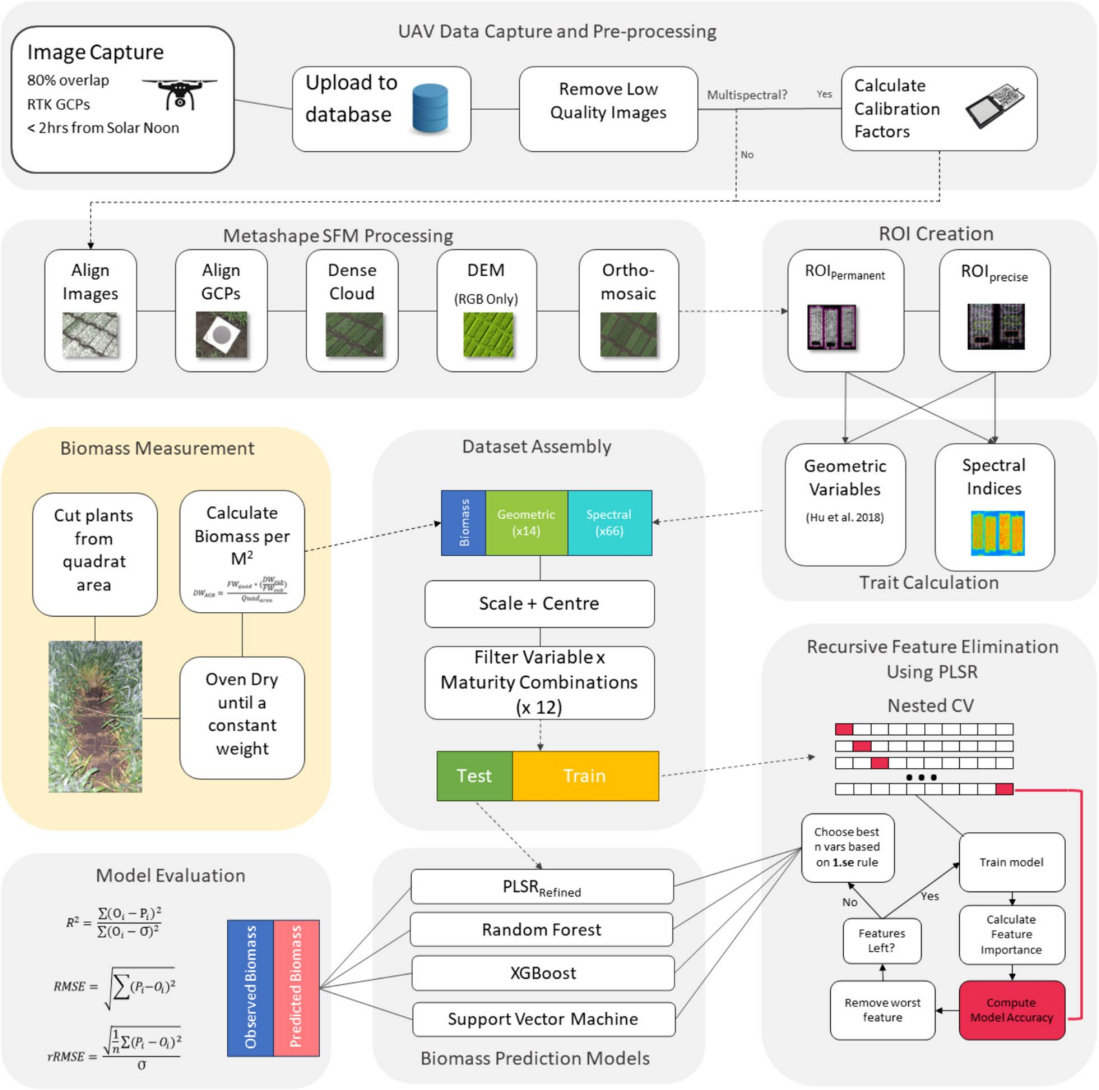
Overall workflow for this study. *PLSR* Partial least squares regression, *GCP* Ground Control Point, *DEM* Digital elevation model, *ROI* Region of Interest, *XGBoost* Extreme Gradient Boosting

The following section was performed using the Caret Package [34] in R Studio 3.0, and the R programming language version 4.2.3 and an overview of the workflow used in this study can be seen in Fig. 4. The dataset was spilt into two parts: 80% as the training set and 20% as an independent test set. This was performed using stratified sampling so that each experiment was represented equally within both the training and test sets. The different combinations of broad growth-stages (i.e., vegetative, flowering, grain-filling, whole season) and predictive variables (i.e., spectral traits, geometric traits, spectral + geometric traits) resulted in 12 different combinations of broad growth-stages and features. Prior to the recursive feature selection using PLSR outlined below, for each of these combinations of features un-supervised filtering of variables based on pairwise correlations was performed to remove highly correlated variables using a correlation coefficient of 0.95 This final number of input parameters for each dataset can be seen in Table S1.

Supervised feature selection was performed on each of the 12 Broad Growth-stage x variable subset groups by recursive feature elimination using a nested cross-validation approach, as per [1]. PLSR was used as the base-learner for this purpose with the number of components in the PLSR model tuned using tenfold cross-validation, and a grid containing 1:the number of features available for that dataset. Each dataset was resampled 30 times into training and test sets with an 80:20 split. For each resample, a PLSR model was initially trained using all features, then, from the set of original features, those with the lowest importance values were removed iteratively until only one feature remained in the model. Feature importance was calculated using the varImp function in caret, which is computed according to the weighted sums of absolute coefficients for each input variable [34]. After each iteration, feature importance was re-calculated, and performance was assessed on the corresponding validation set. The results of the 30 resamples were then aggregated to obtain a performance profile over the feature subset sizes and robust feature importance rankings. The optimal subset size was chosen by selecting the simplest model (with the lowest number of components) whose RMSE value was within 1 standard error of the absolute best model with the lowest RMSE value [5]. This process resulted in 12 sets of features corresponding with the different Broad Growth-stage x variable subsets.The variables selected using this approach served as predictive variables for the models explored below.

[34] PLSR, Random Forest, Support Vector Machine (SVM) and Extreme Gradient Boosting (XGBoost) models were trained for each variable and growth-stage combination using the selected variables based on the RFE feature selection outlined above For an overview of these models refer to the citations provided in Table 3. Each of these models was trained on the training set using a tuning grid of model specific hyperparameters, and K-fold cross validation with 10 folds and 10 repeats (see Table 3 for an overview of model-specific hyperparameters). The average of each individual fold x repeat performance using k-fold validation was calculated as the mean performance of a specific model to reduce the prediction bias caused from random sampling in this small dataset. To decide upon optimal model performance, Root Mean Squared Error (RMSE) was used, while the coefficient of determination (R^2^), and Relative Root Mean Squared Error (rRMSE) was calculated using the R Package Metrica [10].

3 $$R^{2} = \frac{{\sum \left( {{\text{O}}_{i} - {\text{P}}_{i} } \right)^{2} }}{{\sum \left( {{\text{O}}_{i} - {\overline{\text{O}}}} \right)^{2} { }}}$$

4 $$RMSE = \sqrt {\sum \left( {P_{i} - O_{i} } \right)^{2} }$$

5 $$rRMSE = \frac{{\sqrt {\frac{1}{n}\sum \left( {P_{i} - O_{i} } \right)^{2} } }}{{{\overline{\text{O}}}}}$$

where *O*_*i*_ is the observed value for the *i*^*th*^ observation, *P*_*i*_ is the predicted value for the* i*^*th*^ observation, *n* is the total number of observations, and *O*^¯^ is the mean value of all observations.

**Table 3:** Overview of hyperparameters used in cross-validation for each model

Model	Tuning parameter	Min value	Max value	R package	Citation
PLSR	Ncomp	1	Nvars^a^	PLS	Wehrens and Mevik [45]
Random Forest	Mtry	1	Nvars^a^	Ranger	Wright and Ziegler [74]
	Min. Node Size	5			
	Split Rule	Variance			
SVM	Degree	1	2	svmPoly	Kuhn [34]
	C	100	1000		
	Scale	0.0001	0.01		
XGBoost	Nrounds	20	40	xgbTree	Chen et al. [7]
	Max Depth	9	10		
	Eta	0.1	0.3		
	Gamma	0.7	0.9		
	Colsample by tree	0.7	1		

^a^*Nvars* the number of input variables for that model

### Within-season repeatability

The R Package SpATS was used to fit a spatial model for the predictions of each prediction model [57]. SpATS uses restricted log-likelihood (REML) to estimate variance components in the model, and accounts for spatial trends using 2-dimensional p-splines with anisotropic penalties, implemented through the Separation of Anisotropic Penalties (SAP) algorithm [56]. This approach incorporates experimental rows and columns to model the spatial or environmental effect as a two-dimensional penalized tensor-product of marginal B-Spline basis functions.

Genotypic variance ($\sigma G$) in SpATS is estimated following [48], where genetic effects are treated as random effects. Within season repeatability is calculated using effective dimensions, defined as the trace of the corresponding hat matrix, reflecting the contribution of each model component to the phenotypic variation. This approach uses the following equation:

6 $$w^{2} = \frac{{ED_{g} }}{{m_{g} - {\upzeta }\_{\text{g}}}}$$

where $E{D}_{g}$ is the effective dimension for the genetic component, ${m}_{g}$ is the total number of observations, and ζg​ is the shrinkage factor.

## Results

### Predictive variable selection

**Fig. 5:**
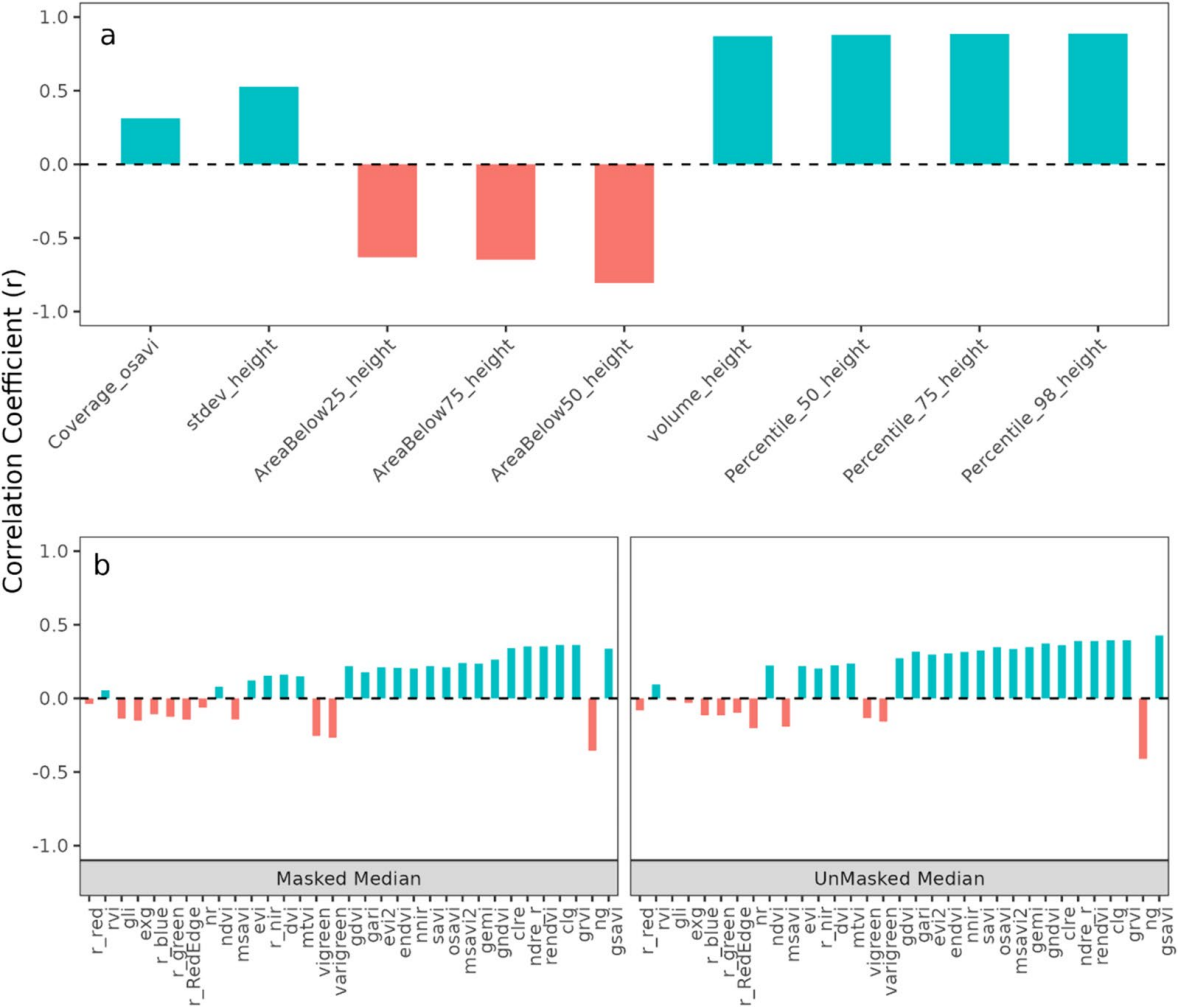
Correlation coefficients (r) of **a** the 9 Geometric variables with dry biomass and **b** both the masked and unmasked median values of spectral traits

The correlation coefficients of the 9 geometric variables as well as 66 median values of spectral variables with DW_AGB_ across all growth stages are illustrated in Fig. 5. These values were calculated on the entire dataset. For geometric traits, the weakest correlation was found for OSAVI canopy coverage. The different percentiles of height each had a positive correlation with DW_AGB_, whereas the area below a particular percentile of height had a negative correlation with DW_AGB_. For spectral traits, the median value of each trait provided the strongest correlation with DW_AGB_, so the sum and mean were disregarded for the analysis. Since masked and unmasked spectral traits provide information that relates to different aspects of the crop canopy and surrounding soil, we decided to use both the masked and unmasked median values in the prediction models. Overall, geometric variables displayed the strongest correlation with DW_AGB_, with 50th, 75th,98th percentile heights each having the greatest correlations (r 0.87–0.88). Meanwhile, spectral traits had a markedly lower correlation with DW_AGB_, with the strongest variable, GSAVI having an r value of 0.43.

From the pairwise correlations seen in Fig. S3 (which utilized all geometric variables (9) and spectral variables (66)), a high degree of co-linearity was apparent amongst spectral traits and certain geometric traits across all growth stages (see Fig. S3). s.. For the four models where geometric and spectral traits were combined, the optimal set of variables always included both spectral traits and geometric traits, however overall, geometric traits were consistently ranked as being the most important, apart from at grain-fill, where red-edge reflectance was ranked as the most important variable (see Table S2). Canopy Volume, ranked as most important geometric trait for all growth-stages combined, vegetative and flowering stages, while at grain-fill, canopy coverage (%) was the most important variable. For spectral traits, rankings varied more considerably, and the overall number of variables chosen was also greater than for geometric variables.

**Fig. 6:**
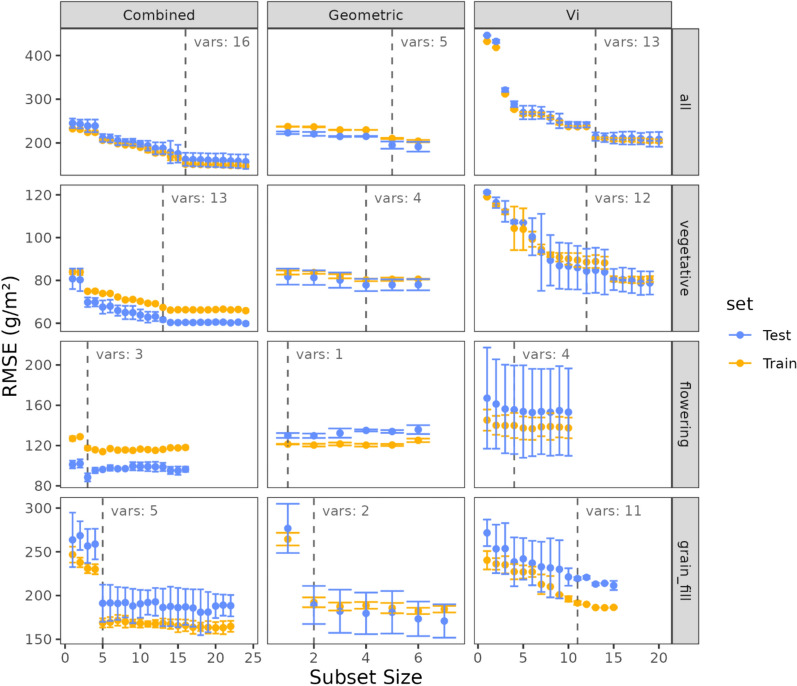
Results of the Feature selection method using recursive feature elimination (RFE), on the x axis is the subset of features, and the y axis is the RMSE (g/m^2^) achieved using nested cross validation. Error bars represent the standard deviation of the RMSE, while points represent the mean RMSE. The Vertical dashed line represents the optimal subset, chosen using the 1.se rule, with the final number of variables indicated in the label

The results of the RFE nested cross validation performance is illustrated in Fig. 6. The optimal RMSE of the underlying PLSR models varied depending on the variable x broad growth-stage grouping investigated. A similar pattern for each of the broad growth-stage groupings was found, where spectral models had the highest RMSE, geometric models had slightly lower RMSE and combined geometric and spectral models had the lowest RMSE. When investigating all maturities combined, the optimal RMSE ranged from 202.8 g/m^2^ using 13 variables when using spectral traits alone, to 208.27 g/m^2^ when using 5 geometric variables alone, and was most accurate when using both geometric and spectral traits combined (147.77 g/m^2^ using 16 variables). For vegetative models, RMSE ranged from 64.4 to 81.24 g/m^2^ when using combined and geometric traits respectively, with the number of chosen variables ranging from 4 (geometric model) to 14 (combined model). For flowering time models, RMSE ranged from 112.89 to 149.44 g/m^2^ when using Combined and Spectral traits respectively, with the number of chosen variables ranging from 1 (geometric model) to 3 (combined model). This stage resulted in the smallest number of chosen variables. For grain-fill models, RMSE ranged from 165.43 to 182.02 g/m^2^ when using Combined and Spectral traits respectively, with the number of chosen variables ranging from 2 (geometric model) to 14 (spectral model).

### Comparison of ML models for biomass prediction

**Fig. 7:**
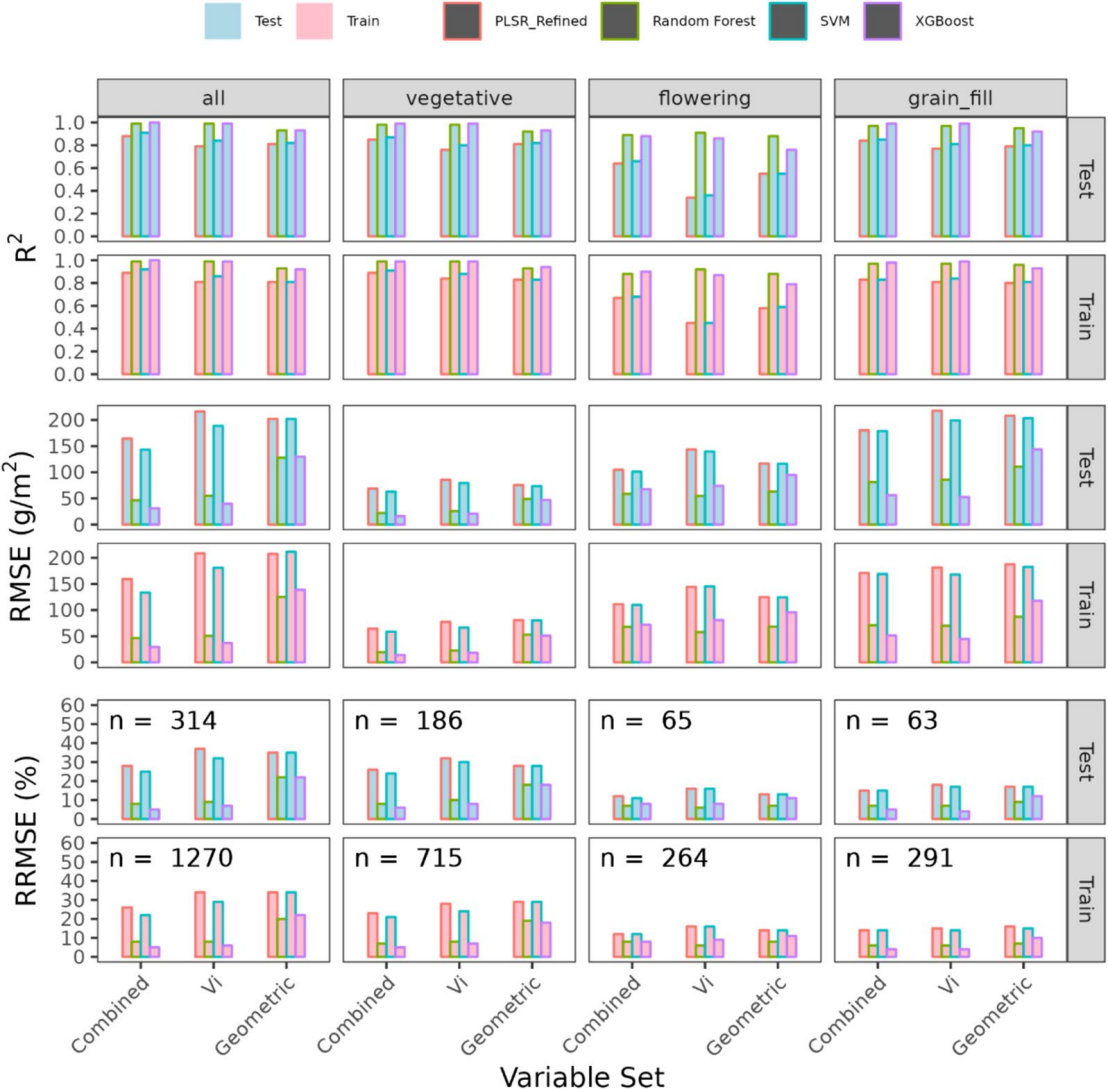
Performance metrics for the prediction of DW_AGB_ (g/m^2^) on the training and test set using the permanent ROI. Each horizontal facet represents the three different metrics used to evaluate model performance: R^2^, RMSE and rRMSE, while vertical facets represent the growth-stage stage at which the models were trained (vegetative, flowering, grain-fill, all (all maturities combined). The X axis includes the three different trait combinations, Geometric + Spectral (Combined), Geometric and Vi. The colour of each bar represents the respective model used to predict DWAGB, and the label represents the number of samples for a particular Growth-stage group

Depending upon the broad growth-stage at which models were trained, models varied in their performance on the independent test set (see Fig. 7). In general, for any specific growth stage, the best model was always obtained using a combination of geometric and spectral variables. For the entire season, the XGBoost model exhibited the highest accuracy, with an RMSE of 31.2 g/m^2^ on the test set and 29.47 g/m^2^ on the training set. The RF model followed with an RMSE of 46.39 g/m^2^ on the test set and 46.6 g/m^2^ on the training set. In contrast, the PLSR_Refined model was the least accurate, with RMSE values of 164.45 g/m^2^ and 159.36 g/m^2^ for the test and train sets, respectively.

At the vegetative stage, the XGBoost model again demonstrated superior performance with RMSE values of 16.14 g/m^2^ (test) and 14.05 g/m^2^ (train). The RF model was slightly less accurate, with RMSEs of 22.11 g/m^2^ (test) and 19.45 g/m^2^ (train). The PLSR_Refined model had higher RMSE values of 68.76 g/m^2^ (test) and 64.51 g/m^2^ (train), indicating lower performance. During the flowering stage, the RF model showed robust accuracy, achieving RMSEs of 54.69 g/m^2^ on the test set and 57.84 g/m^2^ on the training set. XGBoost was comparable, with RMSE values of 67.74 g/m^2^ (test) and 72.11 g/m^2^ (train). Conversely, the PLSR_Refined model's performance was poorer, with RMSEs of 104.9 g/m^2^ (test) and 111.33 g/m^2^ (train). In the grain-fill stage, the XGBoost model maintained high accuracy, with RMSEs of 56.28 g/m^2^ (test) and 51.47 g/m^2^ (train). The RF model followed, with RMSEs of 81.31 g/m^2^ (test) and 70.94 g/m^2^ (train). The PLSR_Refined model, however, lagged with RMSE values of 180.22 g/m^2^ (test) and 171.02 g/m^2^ (train).

When comparing the use of spectral traits, geometric traits, and combined spectral and geometric traits, several general trends emerged. Models using combined spectral and geometric traits consistently outperformed those using only spectral or geometric traits across all stages. For instance, the combined trait models often achieved lower RMSE values, indicating higher predictive accuracy. Specifically, at the vegetative stage, the RF model with combined traits had an RMSE of 22.11 g/m^2^, compared to 48.99 g/m^2^ for geometric traits and 25.79 g/m^2^ for spectral traits.

Spectral traits alone generally provided better performance than geometric traits alone but were still inferior to the combined approach. For example, during the flowering stage, the RF model using spectral traits had an RMSE of 54.69 g/m^2^, while the geometric traits model had an RMSE of 63.04 g/m^2^. Similarly, at the grain-fill stage, spectral traits models showed an RMSE of 85.77 g/m^2^, compared to 110.72 g/m^2^ for geometric traits.

### Permanent ROI vs. Precise ROI

**Fig. 8:**
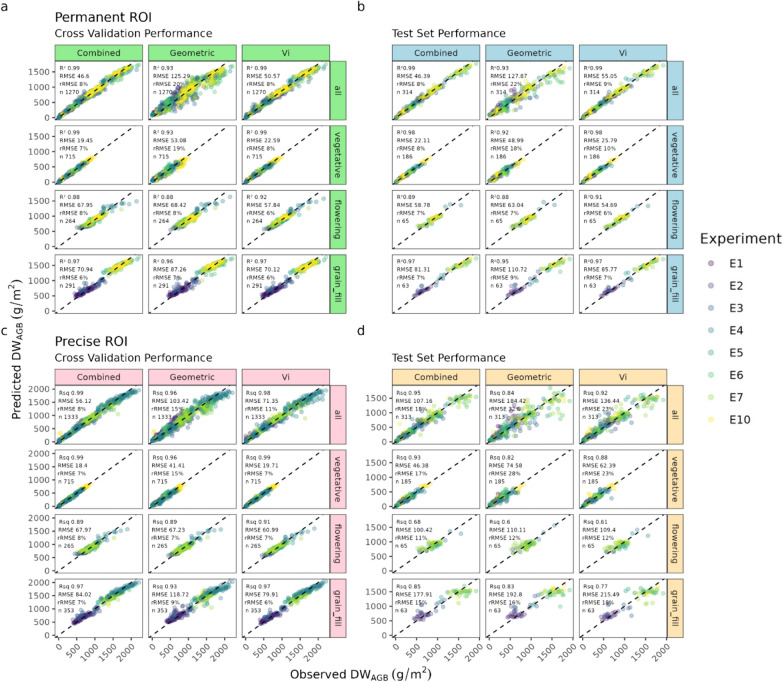
Observed versus predicted *DW*_*AGB*_ on the **permanent ROI**, when looking at **a** Cross validation training set and **b** the independent test set and for the **precise ROI** when looking at **c** Cross-validation performance and **d** Test set performance, for random forest (RF) models trained on combinations of variable types (geometric, spectral, geometric + spectral (Combined)) and different growth-stage combinations (vegetative, flowering, grain-fill, all maturities combined)

While XGBoost models provided the greatest accuracy when using a combination of geometric and spectral traits, we used RF models for the following section, given their similar performance, but faster training times in comparison to XGBoost. In addition to the existing RF models, we trained 12 additional models (on the same combination of 4 maturities and 3 trait combination) using the traits measured from the precise location where biomass cuts were taken (ROI_precise_). Model performance when comparing the ROIs can be seen in Fig. 8, which illustrates that overall, the differences in RMSE between ROI_precise_ and ROI_permanent_ were negligible, although we do see an increase in overall error on ROI_precise_ when examining the test set performance. For example, when using training data from entire season with combined variables, the ROI_permanent_ model had slightly higher accuracy (RMSE 46.6 g/m^2^) than the ROI_permanent_ model (RMSE 56.12 g/m^2^. The largest differences between the ROI types were found when using only geometric traits across the entire season where ROI_precise_ had a RMSE 20.78 g/m^2^ higher than ROI_permanent_. Similarly, across the entire season using spectral traits only, the difference in RMSE was 21.87 g/m^2^, with ROI_permanent_ having the higher accuracy.

### Exploring model generalizability versus specificity

**Fig. 9:**
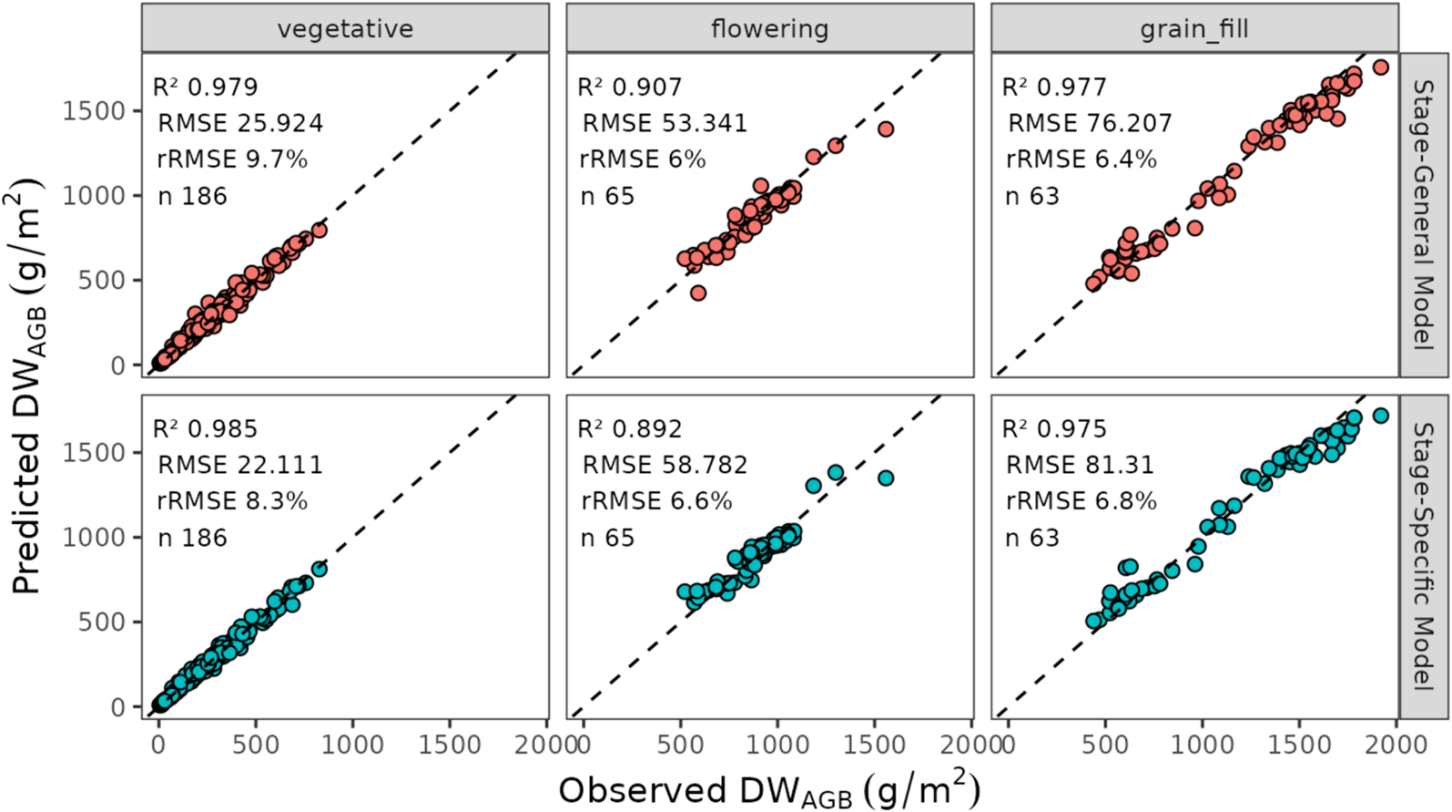
A comparison of biomass prediction on the test set at different growth stages using RF regression using **a** a single model trained across all maturities, and **b** individual models trained only on biomass samples taken from that growth stage

In the exploration of model generalizability, we compared the performance of models trained from data varying in growing stages or experiments (all models using RF with combined geometric and spectral variables). Figure 9 illustrates the prediction accuracy at specific growth stages when using a general model trained on data across all stages (stage-general model) and specific models trained only using data from a specific stage (stage-specific model). The analysis indicates that the optimal model type varies depending on the growth stage. At the vegetative stage, the stage-specific model outperformed the stage-general model with an RMSE of 22.111 g/m^2^ compared to 25.924 g/m^2^. At the flowering stage, the stage-general model had a slightly lower RMSE of 53.341 g/m^2^ compared to 58.782 g/m^2^ for the stage-specific model. Similarly, at the grain-fill stage, the stage-general model achieved an RMSE of 76.207 g/m^2^, whereas the stage-specific model had an RMSE of 81.31 g/m^2^. These results suggest that while stage-specific models can provide more accurate predictions at certain stages, the stage-general model may offer better overall performance at other stages, highlighting the need for tailored modelling approaches depending on the specific growth stage being analysed.

**Fig. 10:**
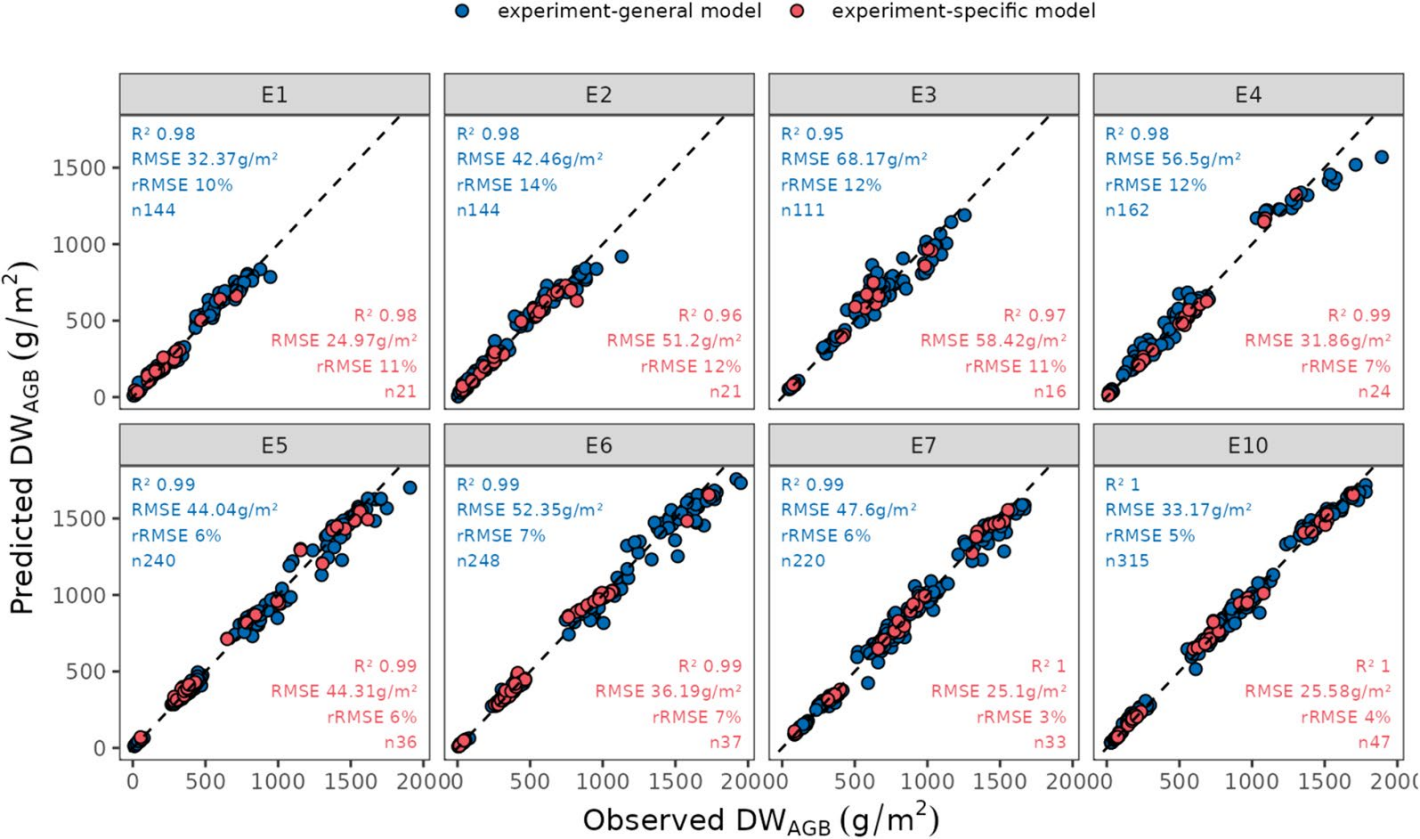
Observed versus predicted DW_AGB_ using a Random Forest (RF) model trained on a combination of Spectral traits and Geometric vars, trained on **a** ground truth data from all experiments, (‘experiment-general model’ shown in blue) and **b** ground truth data for a particular experiment (‘experiment-specific model’ shown in red)

In addition to growth stages, we trained different models using data based on experiments, i.e., a general model (experiment-general model) trained on data from all experiments, and individual models (experiment-specific models) trained only on data from specific experiments. The observed versus predicted biomass results are illustrated in Fig. 10. Among the experiments, E1, E3, E4, E7, and E10 showed significant improvements in accuracy when using experiment-specific models over the experiment-general model. For example, in E4, the RMSE decreased from 56.59 g/m^2^ in the experiment-general model to 31.86 g/m^2^ in the experiment-specific model, showing the greatest benefit from having a specific model with a decrease in RMSE of 24.73 g/m^2^. In E2 and E5, the experiment-general model outperformed the experiment-specific models, with RMSEs decreasing from 51.2 to 42.46 g/m^2^ in E2 and remaining nearly the same in E5 (44.13 g/m^2^ vs. 44.04 g/m^2^). For experiments E6 and E10, the differences in RMSE were smaller but still indicated improved performance with experiment-specific models. These results suggest that while the experiment-general model can perform well, experiment-specific models often provide better accuracy, likely due to the ability to capture subtle differences in experimental conditions.

We also examined the prediction accuracy of the RF model for each individual experiment x DW_AGB_ cut. The model trained on combined geometric + spectral variables was able to predict DW_AGB_ with relatively high accuracy across growth stages and experiments. A general increase in RMSE can be seen as cut numbers and crop-growth-stage increased (i.e. RMSE at cut 1 ranged from 4.4 to 9.16 g/m^2^ and increased to 47.94–89.07 g/m^2^ by cut 5. At the same time, the relative error remained stable, and saw a decrease as cut numbers increased (i.e. rRMSE ranged from 6 to 29% at cut 1, while at cut 5 it ranged from 3 to 6%).

### Within season repeatability biomass prediction models

**Fig. 11:**
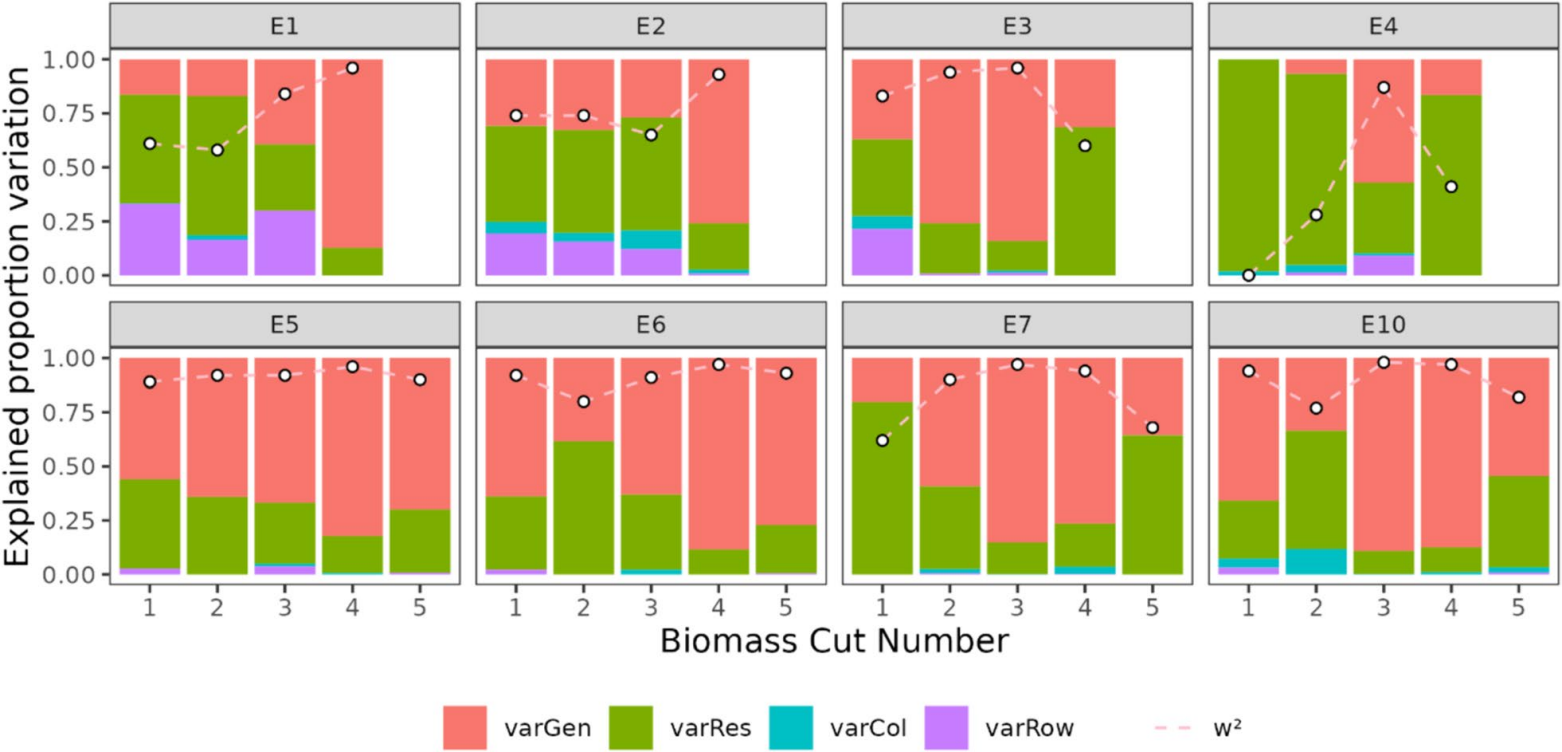
Relationship between biomass cut number and proportion of error variances of predicted biomass from the RF model trained on geometric + spectral indices across all dates and experiments. The x axis represents the biomass cut number and the y axis represents the variation for each respective variance component. White dots represent the within season repeatability (w^2^) for predicted DW_AGB_ at that timepoint

A significant difference in w^2^ was identified across different experiments (p < 0.05), with no apparent relationship between w^2^ and growth-stage. For V_G_, no relationship with the experiment was found, but a significant relationship with growth-stage was observed (p < 0.01). In the context of V_R_, an interaction effect was detected between growth-stage and experiment (p < 0.001). Specifically, experiments 4 and 7 showed higher V_R_ for predicted biomass, while Experiments 3, 5, 6, and 7 reported increases in V_R_ during the grain-fill stage. Conversely, E4 and E7 exhibited lower V_R_ during the vegetative stage. The temporal dynamics of w^2^ based on the predictions from the RF model trained on data points from all maturities and geometric + spectral traits are illustrated in Fig. 11.

## Discussion

The significant increase in interest related to UAV based high throughput phenotyping is partly a result of the need for improved throughput and selection accuracy in breeding programs and NVTs [2, 6]. While the accurate prediction of biomass using HTP platforms has been demonstrated for a range of agricultural crops [17], several practical details with potential to optimize biomass prediction results have yet to be discussed. HTP platforms generate large datasets and given the vast number of possible traits that can be calculated using multispectral and RGB sensors [2], the need for biological interpretability in predictive models is an essential task. Depending on the crop stage at which prediction is made, the ranking of various traits will vary, influencing the type of sensor that is used, and the traits that are necessary to be used.

### Variable importance differs based on growth-stage and sensor type

The importance of using a combination of spectral and Geometric traits in predicting biomass at various growth stages was shown in this study by both the variable selection strategy, and the prediction results from different ML models. Predictions had lower accuracy when models used only geometric variables or spectral traits compared to when they were combined. For models across all maturities, geometric traits ranked as having the greatest importance after recursive feature elimination, however, spectral traits clearly added additional information that improved overall performance. These results reflect recent studies that combine canopy height with spectral traits to predict DW_AGB_. Canopy volume was consistently the first trait selected, however, more complex traits related to the area below a given percentile of height, the standard deviation of height and canopy coverage were also chosen. Canopy Volume has been identified in other literature [41] as an important trait for biomass prediction. Further studies that compare canopy surface provided by RGB cameras, to LIDAR derived volume could help to identify differences in actual biovolume to further improve model accuarcy [72].

When investigating models that used a combination of geometric and spectral traits, changes in the chosen variables may reflect the dynamic relationship between geometric and absorptive/ reflective properties of the canopy and the physiological state of the crop. At vegetative stage, vegetation indices related to canopy greenness (i.e. clg, ng) were highly favoured by the variable selection method. Corti et al. [11] found that spectral traits related to canopy greenness had the strongest correlations with DW_AGB_ across multiple species. Canopy Volume the most important variable at this stage. In contrast, at flowering and grain-fill stages, vegetative indices contributed a smaller proportion to the final models. However, for the spectral indices that were chosen, Red Edge reflectance and traits related to canopy greenness (i.e., NG & masked VARIGREEN) were consistently chosen, which may be due to the relationship between the Red Edge band and the rate and onset of canopy senescence (and consequent photosynthetic function) between different varieties [1].

This study highlighted that combining geometric and spectral traits consistently led to improvements in prediction accuracy. This supports similar studies which found that sensor fusion can provide more comprehensive information surrounding canopy characteristics [11, 14, 50] than individual sensors alone. At the same time, our comparison of spectral and geometric traits highlighted that geometric traits were more closely correlated with canopy biomass at all growth stages. Given both the ubiquity and low cost of modern RGB UAVs, these results indicate RGB UAVs alone may provide a low-cost solution to biomass monitoring in the field through the calculation of geometric traits. It is considered more difficult to obtain consistent results with Multispectral imaging (MS) given the sensitivity to lighting changes and the need for radiometric calibration. While in this study spectral traits (OSAVI) were used to derive Coverage %, the use of classification methods on pixels of RGB imagery, would mean that ‘geometric traits’ could entirely come from an RGB sensor [24, 40].

### Phenology-based classification of growth stages

In this study, the objective of classifying broad growth stages into vegetative, flowering, and grain filling phases was to account for the variation in both structural characteristics and spectral response throughout the development of the canopy. This classification aimed to address the changes that occur as heads emerge (flowering phase) and as they mature and the canopy colour changes, marking the onset of rapid grain filling (grain filling phase). In our study, the vegetative phase was defined to end at Zadok's score of 50, which indicates that 50% of the plants in a plot have at least one awn appearing. The flowering phase was considered to end at Zadok's score of 70, when anthesis is completed on 50% of the plants, signifying that grain development has commenced in many spikelets. We considered further dividing the vegetative phase by introducing a threshold approximately halfway between stem elongation and booting.

However, this subdivision did not enhance the prediction accuracy for the resulting phases. We recognize that with a larger dataset, it might be possible to better optimize the structural and spectral parameters for biomass estimation. This could potentially involve adding an additional phase, such as late vegetative, or adjusting the existing phases to better reflect the relationships between remotely sensed proxies and biomass. A more refined approach could involve developing a generic biomass prediction model, which could then be adjusted using a phenology-derived parameter to optimize predictions continuously across the growth stages.

### Comparison of permanent and precise ROI

While considerable work has been dedicated to the task of ROI generation, as highlighted by [67], the alignment of the ROI with the actual site of ground-truth measurement has not been extensively tested. Through analysis in this work, we found ROI_precise_ (corresponding to the actual locations of biomass cuts) demonstrated accuracy comparable to ROI_permanent_ despite utilization of set of variables across all growth stages. However, variations were noted at different growth stages, and when analysing geometric and spectral traits independently. The discrepancies in model performance between the ROIs are thought to be related to spatial variability within the plot and the distinct characteristics of the areas where ROIs were established. These observations suggest the need for additional research into alternative ROI selection methods and to understand the factors influencing the differences in model performance between ROIs. ROI_precise_ may offer superior performance over ROI_permanent_ particularly in scenarios where within-plot heterogeneity is pronounced.

### Model generalizability

**Fig. 12:**
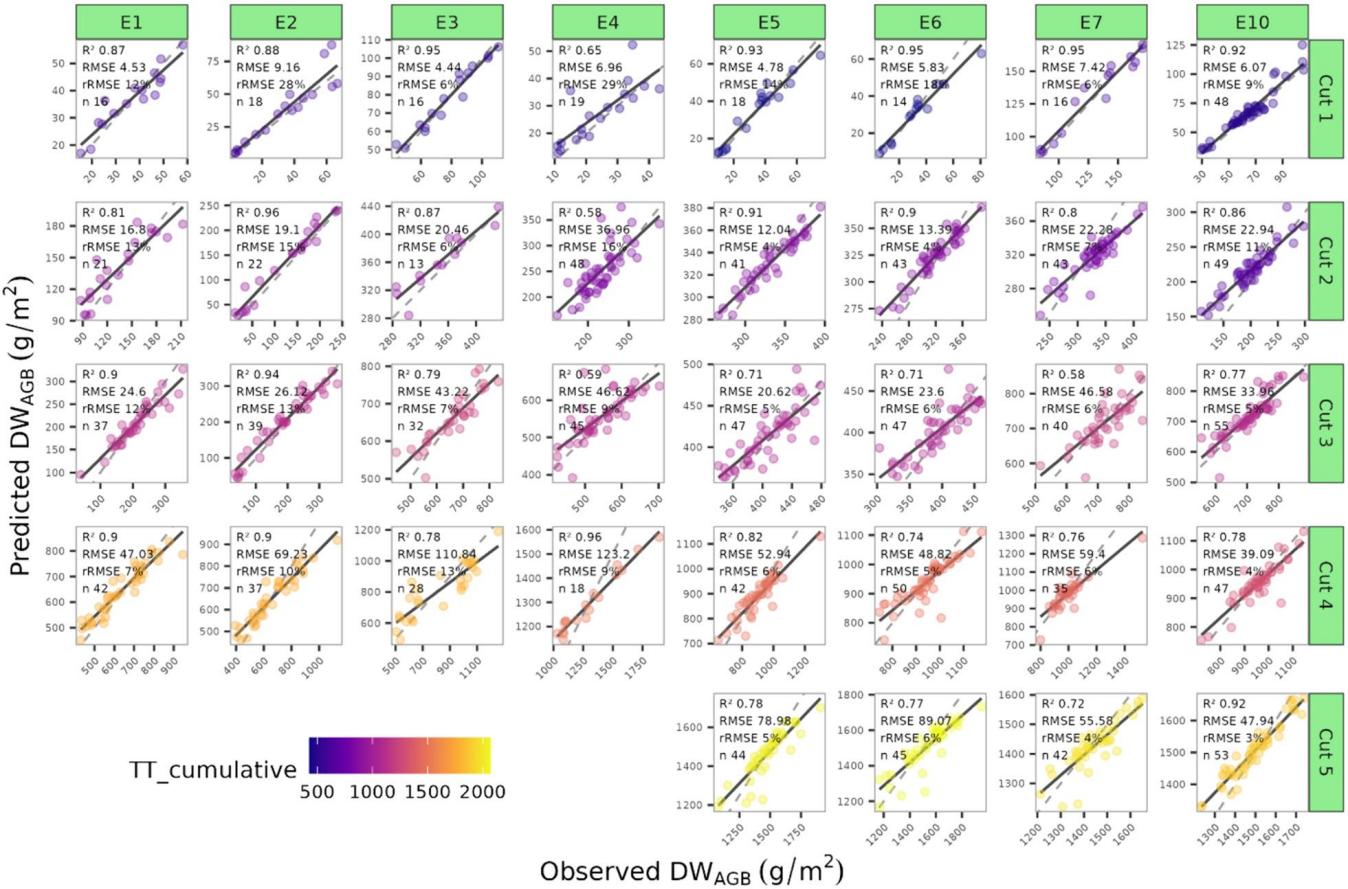
Observed versus predicted DW_AGB_ (g/m^2^) using the Random Forest model trained using geometric and spectral variables on the cross-validated training dataset. Vertical facets represent the different experiments in the study and the horizontal facets represent the DW_AGB_ cuts in order. Point colours represent the cumulative thermal time (TT_cumulative_). Metrics for each cut x experiment are shown in each facet. For test set performance see \* MERGEFORMAT Fig. S4

A key aim in a variety testing situations is the scaling up of predictions to encompass multiple experiments and time points. However, a significant portion of the literature in this field focuses on testing biomass prediction accuracy using a relatively small number of samples from single trials. Our strategy was to evaluate the accuracy of predictions with models trained using the same traits and approaches but distinguishing between specific experiments or growth stages and general models trained on a more comprehensive dataset. Our work indicated that models tailored to a particular growth stage or experiment exhibited comparable (in the case of broad growth-stage) or slightly higher (in the case of specific experiments) accuracy compared to those employing a general approach, presumably because specific models capture specific information that better represents those circumstances. However, the general models still provide adequate predictions across growth stages (Fig. 9), experiments (Fig. 10), and at individual time-points in experiments (Fig. 12) indicating the possibility of using a generic model to make predictions under a wide range of conditions.

### Breeding and variety selection

The findings from the study, particularly those related to biomass prediction and the importance of various variables at different growth stages, can significantly influence breeding decisions and variety selection in wheat crops. w^2^, a key parameter in breeding for trait improvement, was explored in the study, revealing variations across different experiments and maturities. The observed w^2^ can provide insights into the genetic control of the traits under investigation, thereby guiding breeders in selecting varieties that not only exhibit desirable traits but also have a higher probability of passing these traits to subsequent generations. It should be noted that this study focused on a sample of ‘elite’ germplasm, and future research should investigate whether w^2^ is as high for earlier stage breeding material. This study was also limited by the fact that ground-truth DW_AGB_ w^2^ or broad-sense heritability (H^2^) could not be calculated, since only a single replicate was physically sampled at one time. A comparison of the ground-truth w^2^ to HTP derived proxy traits is a key step in determining the optimal timing of sampling, as it defines the ceiling by which the accuracy of remotely-sensed traits can be estimated. This is an important area for future research that requires extra attention.

Since w^2^ is calculated based on the ratio of V_G_ to the total variance (V_G_ + V_R_), to achieve high w^2^, the ratio of V_G_ to V_R_ must also be high. The significant relationship between both V_G_ and growth-stage and V_R_ and growth-stage * experiment means that while w^2^ can be highest at the latest growth stages, the risk of increased V_R_ due to experiment level factors (i.e., lodging or pest damage) can increase. In our experiments, while not significantly different from other growth stages, the growth stage at which mean w^2^ across experiments was highest, was during the flowering stage, which may be a result of this simultaneous increase of V_G_ and V_R_. This may be due to the presence of larger differences among reflectance of genotypes as the differentially reach new phenological stages. This study was limited by the number of locations and years in which testing occurred, and as such, there is a need to investigate the relationship between V_G_ and V_R_ in a wider set of environments to examine whether the timing of measurement can be optimized to maximize w^2^.

### Limitations and future research directions

While the study provides valuable insights into biomass prediction using UAV-based high-throughput phenotyping, several limitations warrant acknowledgment and consideration. One potential limitation is related to the geographical and environmental specificity of the study. The findings and models developed might be highly tailored to the specific environmental conditions and wheat varieties studied, potentially limiting their applicability to different geographical regions, environmental conditions, or wheat varieties. Furthermore, the varieties used in this study were limited to ‘elite’ breeding materials, which potentially lack the range of variation that might be present in earlier-stage breeding trials where selection may be warranted. Future research should also compare the w^2^ and H^2^ of ground-truth samples to the estimated values for DW_AGB_, so as to confirm the genetic-gain, or selection advantage for using HTP derived traits.

In future research, we also recommend exploring the use of parametric models to further analyse the relationships between biomass and selected predictors. This approach would involve selecting a subset of variables that maintains performance close to that of the full model and then fitting a parametric model to these predictors. Such a model would allow for clearer interpretation of the impact of each predictor on biomass at different stages of crop development, enhancing the overall interpretability and robustness of the results.

This methodology, whilst providing a proof of concept for scaling up an empirical calibration for biomass, still requires manual ground-truth measurements for model building. Even taking a small number of biomass samples can be a costly undertaking, especially where multiple experiments and locations are concerned. As such, there is a need to determine an economically viable number of biomass samples that can be taken, whilst still maintaining model accuracy and w^2^. One such approach, as demonstrated by Hu et al. [29] is to take a self-calibration approach, which takes advantage of empirical ground-truth measurements, but optimizes the number of samples necessary to maintain accuracy whilst maintaining low cost. In a situation with 1000 s of plots, you can use a generalized model to estimate biomass and immediately choose a diverse set of plots to measure the biomass (20 to 50 plots) to create an improved model calibration. In addition, the topic of sub-plot selection and analysis warrants further exploration. Underlying within-plot variability is a source of variability that may not be properly accounted for when building the ROI to analyse secondary traits from UAVs. Whilst in experiments with low variability this may not be a major issue, but where variability is high, alternative approaches such as the one proposed in this paper might lead to higher prediction accuracies and w^2^ values.

## Conclusion

This study explored the sensitivity of UAV-based biomass prediction in wheat, exploring the influence of variable type, modelling strategy and sampling location on model accuracy. We utilised robust feature selection, using recursive feature elimination, to identify key features associated with biomass at varying growth stages and using different sensor traits. A combination of RGB and multispectral traits was confirmed to provide the greatest accuracy across growth stages, with canopy height and volume having the greatest importance, but being supplemented by growth-stage specific spectral indices. The comparison of the specific and permanent ROI did not result in significant differences in model accuracy, however this may have been due to the relative homogeneity in experiments, and further investigation into this approach in heterogenous situations may provide greater accuracy. In this case a general model trained across all available data performed comparably with stage-specific and experiment-specific models, highlighting the ability for Machine Learning methods to capture complex relationships in the data. Overall, biomass prediction using UAV offers a non-destructive and scalable alternative to manual measurements, however, the need for careful modelling to demonstrate physiological relevance, and ability to be used for variety selection is still necessary.

### Supplementary Information

Supplementary material 1 Fig. S1 Relationship between the Date of biomass cut and the date of the corresponding UAV flight for each experiment. Point colours represent the trial mean Zadok’s stage at a given timepoint. Fig. S2 Examples of the thresholding methodology that was used in the study. The panels include a true colour image, the OSAVI vegetation index and binary masks after thresholding using Otsu’s method. Table S1 Overview of number of samples included for each Variable and Growth-stage combination assessed in this study, including number of observations in the train set and test set, along with the number of input features included after filtering for co-linearity. Table S2 Overview of selected variables after recursive feature elimination (RFE) for each of the 12 variable x growth stage combinations. Text colour indicates whether a variable is geometric (blue) or spectral (orange). The numbering of the columns indicates the order in which each variable was selected. Table S3 Train and Test performance metrics for each of the Growth-stage x variable set x Model types on the Permanent ROI. Table S4 Abbreviation, calculation, name, and key reference for the Spectral indices used as input to biomass prediction models in this study. Table S5 Calculation of Geometric Variables for use as input variables for biomass prediction models. Table includes the name of the trait, a description of how it was calculated, and a relevant reference. Fig. S3 Correlation Matrix illustrating the relationship between all variables and all timepoints, ordered by the angle of eigenvectors (AOE). Fig. S4 Observed versus predicted DW_AGB_ (g/m^2^) using the Random Forest model trained using geometric and spectral variables on the independent test set. Vertical facets represent the different experiments in the study and the horizontal facets represent the DW_AGB_ cuts in numeric order. Point colours represent the cumulative thermal time (TT_cumulative_). Metrics for each cut x experiment are shown in each facet.

## Data Availability

The datasets generated and/or analysed during the current study are available from the corresponding author on reasonable request. This ensures the accessibility of data while maintaining the necessary controls for their ethical and responsible use.

## Code availability

The custom code developed for this study is available from the corresponding author upon reasonable request. This approach ensures that the code can be accessed for legitimate research purposes while maintaining appropriate oversight and adherence to ethical standards in its use and application.

## References

[1] Anderegg J, Yu K, Aasen H, Walter A, Liebisch F, Hund A. Spectral vegetation indices to track senescence dynamics in diverse wheat germplasm. Front Plant Sci. 2020. 10.3389/fpls.2019.01749. 32047504 10.3389/fpls.2019.01749 PMC6997566

[2] Araus JL, Cairns JE. Field high-throughput phenotyping: the new crop breeding frontier. Trends Plant Sci. 2014;19(1):52–61. 10.1016/j.tplants.2013.09.008. 24139902 10.1016/j.tplants.2013.09.008

[3] Baret F, Jacquemoud S, Hanocq J. The soil line concept in remote sensing. Remote Sens Rev. 1993;7(1):65–82. 10.1080/02757259309532166

[4] Bendig J, Bolten A, Bennertz S, Broscheit J, Eichfuss S, Bareth G. Estimating biomass of barley using crop surface models (CSMs) derived from UAV-based RGB imaging. Remote Sens. 2014;6(11):10395–412. 10.3390/rs61110395. 10.3390/rs61110395

[5] Breiman L, Friedman J, Olshen RA, Stone CJ (1984) Classification and Regression Trees (1st ed.). Chapman and Hall/CRC. 10.1201/9781315139470

[6] Cabrera-Bosquet L, Crossa J, von Zitzewitz J, Serret MD, Araus JL. High-throughput phenotyping and genomic selection: the frontiers of crop breeding converge. J Integr Plant Biol. 2012;54(5):312–20. 10.1111/j.1744-7909.2012.01116.x. 22420640 10.1111/j.1744-7909.2012.01116.x

[7] Chen T, He T, Benesty M, Khotilovich V, Tang Y, Cho H, Chen K, Mitchell R, Cano I, Zhou T. Xgboost: extreme gradient boosting. R package version 0.4-2. 2015; 1(4): 1–4.

[8] Comstock RE. Quantitative genetics and the design of breeding programs. In E. Pollak (Eds). Proc. Int. Conf. on Quant. Genet., Ames, Iowa. 16–21 Aug. Iowa State University Press. 1977; 705–718.

[9] Coombes N.. DiGGer design search tool in R. 2009. http://nswdpibiom.org/austatgen/software. Accessed 20 Mar 2024

[10] Correndo AA, Rosso LHM, Hernandez CH, Bastos LM, Nieto L, Holzworth D, Ciampitti IA. metrica: an R package to evaluate prediction performance of regression and classification point-forecast models. J Open Sour Softw. 2022;7(79):4655. 10.21105/joss.04655

[11] Corti M, Cavalli D, Cabassi G, Bechini L, Pricca N, Paolo D, Marinoni L, Vigoni A, Degano L, Marino Gallina P. Improved estimation of herbaceous crop aboveground biomass using UAV-derived crop height combined with vegetation indices. Precision Agric. 2023;24(2):587–606. 10.1007/s11119-022-09960-w

[12] Das S, Massey-Reed SR, Mahuika J, Watson J, Cordova C, Otto L, Zhao Y, Chapman S, George-Jaeggli B, Jordan D. A high-throughput phenotyping pipeline for rapid evaluation of morphological and physiological crop traits across large fields. IGARSS 2022–2022 IEEE International Geoscience and Remote Sensing Symposium. 2022.

[13] Deery D, Jimenez-Berni J, Jones H, Sirault X, Furbank R. Proximal remote sensing buggies and potential applications for field-based phenotyping. Agron Basel. 2014;4(3):349–79. 10.3390/agronomy4030349. 10.3390/agronomy4030349

[14] Deery DM, Rebetzke GJ, Jimenez-Berni JA, Condon AG, Smith DJ, Bechaz KM, Bovill WD. Ground-based LiDAR improves phenotypic repeatability of above-ground biomass and crop growth rate in wheat. Plant Phenomics. 2020. 10.34133/2020/8329798. 33313565 10.34133/2020/8329798 PMC7706344

[15] Fitzgerald G, Rodriguez D, Christensen L, Belford R, Sadras V, Clarke T. Spectral and thermal sensing for nitrogen and water status in rainfed and irrigated wheat environments. Precision Agric. 2006;7:233–48. 10.1007/s11119-006-9011-z

[16] Fu Y, Yang G, Song X, Li Z, Xu X, Feng H, Zhao C. Improved estimation of winter wheat aboveground biomass using multiscale textures extracted from UAV-based digital images and hyperspectral feature analysis. Remote Sens. 2021;13(4):581. 10.3390/rs13040581. 10.3390/rs13040581

[17] Furbank RT, Jimenez-Berni JA, George-Jaeggli B, Potgieter AB, Deery DM. Field crop phenomics: enabling breeding for radiation use efficiency and biomass in cereal crops. New Phytol. 2019;223(4):1714–27. 10.1111/nph.15817. 30937909 10.1111/nph.15817

[18] Gill T, Gill SK, Saini DK, Chopra Y, de Koff JP, Sandhu KS. A comprehensive review of high throughput phenotyping and machine learning for plant stress phenotyping. Phenomics. 2022;2(3):156–83. 36939773 10.1007/s43657-022-00048-z PMC9590503

[19] Gitelson AA, Kaufman YJ, Merzlyak MN. Use of a green channel in remote sensing of global vegetation from EOS-MODIS. Remote Sens Environ. 1996;58(3):289–98. 10.1016/S0034-4257(96)00072-7

[20] Gitelson AA, Kaufman YJ, Stark R, Rundquist D. Novel algorithms for remote estimation of vegetation fraction. Remote Sens Environ. 2002;80(1):76–87. 10.1016/S0034-4257(01)00289-9

[21] Gitelson AA, Merzlyak M, Zur Y, Stark R, Gritz U. Non-destructive and remote sensing techniques for estimation of vegetation status. Proceedings of the 3rd European Conference on Precision Agriculture, Montpelier, France. 2001.

[22] Gitelson AA, Viña A, Ciganda V, Rundquist DC, Arkebauer TJ. Remote estimation of canopy chlorophyll content in crops. Geophys Res Lett. 2005. 10.1029/2005GL022688. 10.1029/2005GL022688

[23] Gobron N, Pinty B, Verstraete MM, Widlowski J-L. Advanced vegetation indices optimized for up-coming sensors: design, performance, and applications. IEEE Trans Geosci Remote Sens. 2000;38(6):2489–505. 10.1109/36.885197

[24] Guo W, Zheng B, Duan T, Fukatsu T, Chapman S, Ninomiya S. EasyPCC: benchmark datasets and tools for high-throughput measurement of the plant canopy coverage ratio under field conditions. Sensors. 2017;17(4):798. 28387746 10.3390/s17040798 PMC5422159

[25] Haboudane D, Miller JR, Pattey E, Zarco-Tejada PJ, Strachan IB. Hyperspectral vegetation indices and novel algorithms for predicting green LAI of crop canopies: modeling and validation in the context of precision agriculture. Remote Sens Environ. 2004;90(3):337–52. 10.1016/j.rse.2003.12.013

[26] Hall MA. Correlation-based feature selection for machine learning The University of Waikato. 1999.

[27] Hoefler R, González-Barrios P, Bhatta M, Nunes JA, Berro I, Nalin RS, Borges A, Covarrubias E, Diaz-Garcia L, Quincke M. Do spatial designs outperform classic experimental designs? J Agric Biol Environ Stat. 2020;25(4):523–52. 10.1007/s13253-020-00406-2

[28] Hu P, Chapman SC, Jin H, Guo Y, Zheng B. Comparison of modelling strategies to estimate phenotypic values from an unmanned aerial vehicle with spectral and temporal vegetation indexes. Remote Sens. 2021;13(14):2827. 10.3390/rs13142827

[29] Hu P, Chapman SC, Wang X, Potgieter A, Duan T, Jordan D, Guo Y, Zheng B. Estimation of plant height using a high throughput phenotyping platform based on unmanned aerial vehicle and self-calibration: example for sorghum breeding. Eur J Agron. 2018;95:24–32. 10.1016/j.eja.2018.02.004

[30] Huete AR. A soil-adjusted vegetation index (SAVI). Remote Sens Environ. 1988;25(3):295–309. 10.1016/0034-4257(88)90106-X

[31] Jiang Z, Huete AR, Didan K, Miura T. Development of a two-band enhanced vegetation index without a blue band. Remote Sens Environ. 2008;112(10):3833–45. 10.1016/j.rse.2008.06.006

[32] Jin X, Madec S, Dutartre D, de Solan B, Comar A, Baret F. High-throughput measurements of stem characteristics to estimate ear density and above-ground biomass. Plant Phenomics. 2019. 10.34133/2019/4820305. 33313528 10.34133/2019/4820305 PMC7706336

[33] Jordan CF. Derivation of leaf-area index from quality of light on the forest floor. Ecology. 1969;50(4):663–6. 10.2307/1936256

[34] Kuhn M. Building predictive models in R using the caret package. J Stat Softw. 2008;28:1–26.27774042 10.18637/jss.v028.i05

[35] Kursa MB, Rudnicki WR. Feature selection with the Boruta package. J Stat Softw. 2010;36:1–13. 10.18637/jss.v036.i11

[36] Li J, Shi Y, Veeranampalayam-Sivakumar A-N, Schachtman DP. Elucidating sorghum biomass, nitrogen and chlorophyll contents with spectral and morphological traits derived from unmanned aircraft system. Front Plant Sci. 2018. 10.3389/fpls.2018.01406. 30333843 10.3389/fpls.2018.01406 PMC6176777

[37] Liu HQ, Huete A. A feedback based modification of the NDVI to minimize canopy background and atmospheric noise. IEEE Trans Geosci Remote Sens. 1995;33(2):457–65. 10.1109/TGRS.1995.8746027

[38] Liu Y, Liu S, Li J, Guo X, Wang S, Lu J. Estimating biomass of winter oilseed rape using vegetation indices and texture metrics derived from UAV multispectral images. Comput Electron Agric. 2019;166:105026. 10.1016/j.compag.2019.105026

[39] Lu N, Zhou J, Han Z, Li D, Cao Q, Yao X, Tian Y, Zhu Y, Cao W, Cheng T. Improved estimation of aboveground biomass in wheat from RGB imagery and point cloud data acquired with a low-cost unmanned aerial vehicle system. Plant Methods. 2019;15(1):1–16. 30828356 10.1186/s13007-019-0402-3 PMC6381699

[40] Madec S, Irfan K, Velumani K, Baret F, David E, Daubige G, Samatan LB, Serouart M, Smith D, James C. VegAnn, vegetation annotation of multi-crop RGB images acquired under diverse conditions for segmentation. Sci Data. 2023;10(1):302. 37208401 10.1038/s41597-023-02098-y PMC10199053

[41] Maimaitijiang M, Sagan V, Sidike P, Maimaitiyiming M, Hartling S, Peterson KT, Maw MJ, Shakoor N, Mockler T, Fritschi FB. Vegetation index weighted canopy volume model (CVMVI) for soybean biomass estimation from unmanned aerial system-based RGB imagery. ISPRS J Photogramm Remote Sens. 2019;151:27–41. 10.1016/j.isprsjprs.2019.03.003

[42] Malone B, Stockmann U, Glover M, McLachlan G, Engelhardt S, Tuomi S. Digital soil survey and mapping underpinning inherent and dynamic soil attribute condition assessments. Soil Secur. 2022;6:100048. 10.1016/j.soisec.2022.100048.

[43] Marcial-Pablo MDJ, Gonzalez-Sanchez A, Jimenez-Jimenez SI, Ontiveros-Capurata RE, Ojeda-Bustamante W. Estimation of vegetation fraction using RGB and multispectral images from UAV [Marcial-Pablo2019]. Int J Remote Sens. 2019;40(2):420–38. 10.1080/01431161.2018.1528017

[44] MaxMax. Enhanced Normalized Difference Vegetation Index. 2015. https://www.maxmax.com/endvi.htm. Accessed 20 Mar 2024

[45] Mevik B-H, Wehrens R. The pls package: principal component and partial least squares regression in R. J Stat Softw. 2007;18(2):1–23 10.18637/jss.v018.i02

[46] Mondal S, Dutta S, Crespo-Herrera L, Huerta-Espino J, Braun HJ, Singh RP. Fifty years of semi-dwarf spring wheat breeding at CIMMYT: grain yield progress in optimum, drought and heat stress environments. Field Crop Res. 2020;250:107757. 10.1016/j.fcr.2020.107757

[47] Montes JM, Technow F, Dhillon BS, Mauch F, Melchinger AE. High-throughput non-destructive biomass determination during early plant development in maize under field conditions. Field Crop Res. 2011;121(2):268–73. 10.1016/j.fcr.2010.12.017

[48] Oakey H, Verbyla A, Pitchford W, Cullis B, Kuchel H. Joint modeling of additive and non-additive genetic line effects in single field trials. Theor Appl Genet. 2006;113:809–19. 16896718 10.1007/s00122-006-0333-z

[49] Otsu N. A threshold selection method from gray-level histograms. IEEE Trans Syst Man Cybern. 1979;9(1):62–6. 10.1109/TSMC.1979.4310076

[50] Pauli D, Chapman SC, Bart R, Topp CN, Lawrence-Dill CJ, Poland J, Gore MA. The quest for understanding phenotypic variation via integrated approaches in the field environment. Plant Physiol. 2016;172(2):622–34. 10.1104/pp.16.00592. 27482076 10.1104/pp.16.00592 PMC5047081

[51] Qi J, Chehbouni A, Huete AR, Kerr YH, Sorooshian S. A modified soil adjusted vegetation index. Remote Sens Environ. 1994;48(2):119–26. 10.1016/0034-4257(94)90134-1

[52] Reynolds M, Langridge P. Physiological breeding. Curr Opin Plant Biol. 2016;31:162–71. 10.1016/j.pbi.2016.04.005. 27161822 10.1016/j.pbi.2016.04.005

[53] Reynolds MP, Slafer GA, Foulkes JM, Griffiths S, Murchie EH, Carmo-Silva E, Asseng S, Chapman SC, Sawkins M, Gwyn J. A wiring diagram to integrate physiological traits of wheat yield potential. Nature Food. 2022;3(5):318–24. 37117579 10.1038/s43016-022-00512-z

[54] Richards JA, Richards JA. Remote sensing digital image analysis. Berlin: Springer; 2022. 10.1007/978-3-030-82327-6

[55] Richardson AJ, Wiegand C. Distinguishing vegetation from soil background information. Photogramm Eng Remote Sens. 1977;43(12):1541–52.

[56] Rodriguez-Alvarez MX, Boer M, Eilers P, van Eeuwijk F. Spatial analysis of field trials with splines. 2020. https://cran.r-project.org/web/packages/SpATS/SpATS.pdf

[57] Rodríguez-Álvarez MX, Boer MP, van Eeuwijk FA, Eilers PH. Correcting for spatial heterogeneity in plant breeding experiments with P-splines. Sp Stat. 2018;23:52–71. 10.1016/j.spasta.2017.10.003. 10.1016/j.spasta.2017.10.003

[58] Rouse J, Haas RH, Schell JA, Deering DW. Monitoring vegetation systems in the Great Plains with ERTS. NASA Spec Publ. 1974;351:309.

[59] Roy Choudhury M, Das S, Christopher J, Apan A, Chapman S, Menzies NW, Dang YP. Improving biomass and grain yield prediction of wheat genotypes on sodic soil using integrated high-resolution multispectral, hyperspectral, 3D point cloud, and machine learning techniques. Remote Sens. 2021;13(17):3482. 10.3390/rs13173482

[60] Schmidt P, Hartung J, Rath J, Piepho H-P. Estimating broad-sense heritability with unbalanced data from agricultural cultivar trials. Crop Sci. 2019;59(2):525–36. 10.2135/cropsci2018.06.0376

[61] Sharma P, Leigh L, Chang J, Maimaitijiang M, Caffé M. Above-ground biomass estimation in oats using UAV remote sensing and machine learning. Sensors. 2022;22(2):601. 35062559 10.3390/s22020601 PMC8778966

[62] Sinclair TR, Muchow RC. Radiation use efficiency. Adv Agron. 1999;65:215–65. 10.1016/S0065-2113(08)60914-1

[63] Singh A, Ganapathysubramanian B, Singh AK, Sarkar S. Machine learning for high-throughput stress phenotyping in plants. Trends Plant Sci. 2016;21(2):110–24. 26651918 10.1016/j.tplants.2015.10.015

[64] Smith DT, Potgieter AB, Chapman SC. Scaling up high-throughput phenotyping for abiotic stress selection in the field. Theor Appl Genet. 2021. 10.1007/s00122-021-03864-5. 34076731 10.1007/s00122-021-03864-5

[65] Sripada RP. Determining in-season nitrogen requirements for corn using aerial color-infrared photography. North Carolina State University. 2005.

[66] Toda Y, Kaga A, Kajiya-Kanegae H, Hattori T, Yamaoka S, Okamoto M, Tsujimoto H, Iwata H. Genomic prediction modeling of soybean biomass using UAV-based remote sensing and longitudinal model parameters. Plant Genome. 2021;14(3):e20157. 34595846 10.1002/tpg2.20157 PMC12807238

[67] Tresch L, Mu Y, Itoh A, Kaga A, Taguchi K, Hirafuji M, Ninomiya S, Guo W. Easy MPE: extraction of quality microplot images for UAV-based high-throughput field phenotyping. Plant Phenomics. 2019. 10.34133/2019/2591849. 33313523 10.34133/2019/2591849 PMC7706339

[68] Tucker CJ. Red and photographic infrared linear combinations for monitoring vegetation. Remote Sens Environ. 1979;8(2):127–50. 10.1016/0034-4257(79)90013-0. 10.1016/0034-4257(79)90013-0

[69] Wang D, Li R, Zhu B, Liu T, Sun C, Guo W. Estimation of wheat plant height and biomass by combining UAV imagery and elevation data. Agriculture. 2022;13(1):9. 10.3390/agriculture13010009

[70] Wang H, Duan Y, Shi Y, Kato Y, Ninomiya S, Guo W. EasyIDP: a python package for intermediate data processing in UAV-based plant phenotyping. Remote Sens. 2021;13(13):2622. 10.3390/rs13132622

[71] Wang T, Crawford MM, Tuinstra MR. A novel transfer learning framework for sorghum biomass prediction using UAV-based remote sensing data and genetic markers. Front Plant Sci. 2023;14:1138479. 37113602 10.3389/fpls.2023.1138479 PMC10126475

[72] Wiering NP, Ehlke NJ, Sheaffer CC. Lidar and RGB image analysis to predict hairy vetch biomass in breeding nurseries. Plant Phenome J. 2019;2(1):1–8. 10.2135/tppj2019.02.0003

[73] Woebbecke DM, Meyer GE, Von Bargen K, Mortensen DA. Color indices for weed identification under various soil, residue, and lighting conditions. Trans ASAE. 1995;38(1):259–69. 10.13031/2013.27838

[74] Wright MN, Ziegler A. Ranger: a fast implementation of random forests for high dimensional data in C++ and R. J Stat Softw. 2017;77(1):1–17. 10.18637/jss.v077.i01. 10.18637/jss.v077.i01

[75] Yoosefzadeh-Najafabadi M, Tulpan D, Eskandari M. Using hybrid artificial intelligence and evolutionary optimization algorithms for estimating soybean yield and fresh biomass using hyperspectral vegetation indices. Remote Sens. 2021;13(13):2555. 10.3390/rs13132555

[76] Yue J, Yang G, Tian Q, Feng H, Xu K, Zhou C. Estimate of winter-wheat above-ground biomass based on UAV ultrahigh-ground-resolution image textures and vegetation indices. ISPRS J Photogramm Remote Sens. 2019;150:226–44. 10.1016/j.isprsjprs.2019.02.022

[77] Zadoks JC, Chang TT, Konzak CF. A decimal code for the growth stages of cereals. Weed Res. 1974;14(6):415–21. 10.1111/j.1365-3180.1974.tb01084.x

[78] Zarco-Tejada PJ, Miller JR, Noland TL, Mohammed GH, Sampson PH. Scaling-up and model inversion methods with narrowband optical indices for chlorophyll content estimation in closed forest canopies with hyperspectral data. IEEE Trans Geosci Remote Sens. 2001;39(7):1491–507. 10.1109/36.934080

[79] Zhang Y, Xia C, Zhang X, Cheng X, Feng G, Wang Y, Gao Q. Estimating the maize biomass by crop height and narrowband vegetation indices derived from UAV-based hyperspectral images. Ecol Ind. 2021;129:107985. 10.1016/j.ecolind.2021.107985

[80] Zheng B, Chenu K, Doherty A, Chapman S. The APSIM-wheat module (7.5 R3008). Agricultural Production Systems Simulator (APSIM) Initiative. 2014; 615. http://www.apsim.info/Portals/0/Documentation/Crops/WheatDocumentation.pdf

