# Supplementary material for: Prediction accuracy and repeatability of UAV based biomass estimation in wheat variety trials as affected by variable type, modelling strategy and sampling location
Source: Plant Methods. 2024 Aug 20;20:129. doi: 10.1186/s13007-024-01236-w (PMC11337646; doi:10.1186/s13007-024-01236-w)
Supplement: Supplementary file 1 — Supplementary material 1 Fig. S1 Relationship between the Date of biomass cut and the date of the corresponding UAV flight for each experiment. Point colours represent the trial mean Zadok’s stage at a given timepoint. Fig. S2 Examples of the thresholding methodology that was used in the study. The panels include a true colour image, the OSAVI vegetation index and binary masks after thresholding using Otsu’s method. Table S1 Overview of number of samples included for each Variable and Growth-stage combination assessed in this study, including number of observations in the train set and test set, along with the number of input features included after filtering for co-linearity. Table S2 Overview of selected variables after recursive feature elimination (RFE) for each of the 12 variable x growth stage combinations. Text colour indicates whether a variable is geometric (blue) or spectral (orange). The numbering of the columns indicates the order in which each variable was selected. Table S3 Train and Test performance metrics for each of the Growth-stage x variable set x Model types on the Permanent ROI. Table S4 Abbreviation, calculation, name, and key reference for the Spectral indices used as input to biomass prediction models in this study. Table S5 Calculation of Geometric Variables for use as input variables for biomass prediction models. Table includes the name of the trait, a description of how it was calculated, and a relevant reference. Fig. S3 Correlation Matrix illustrating the relationship between all variables and all timepoints, ordered by the angle of eigenvectors (AOE). Fig. S4 Observed versus predicted DWAGB (g/m2) using the Random Forest model trained using geometric and spectral variables on the independent test set. Vertical facets represent the different experiments in the study and the horizontal facets represent the DWAGB cuts in numeric order. Point colours represent the cumulative thermal time (TTcumulative). Metrics for each cut x experiment a [file 13007_2024_1236_MOESM1_ESM.docx]

# Appendix 1 – Supplementary information


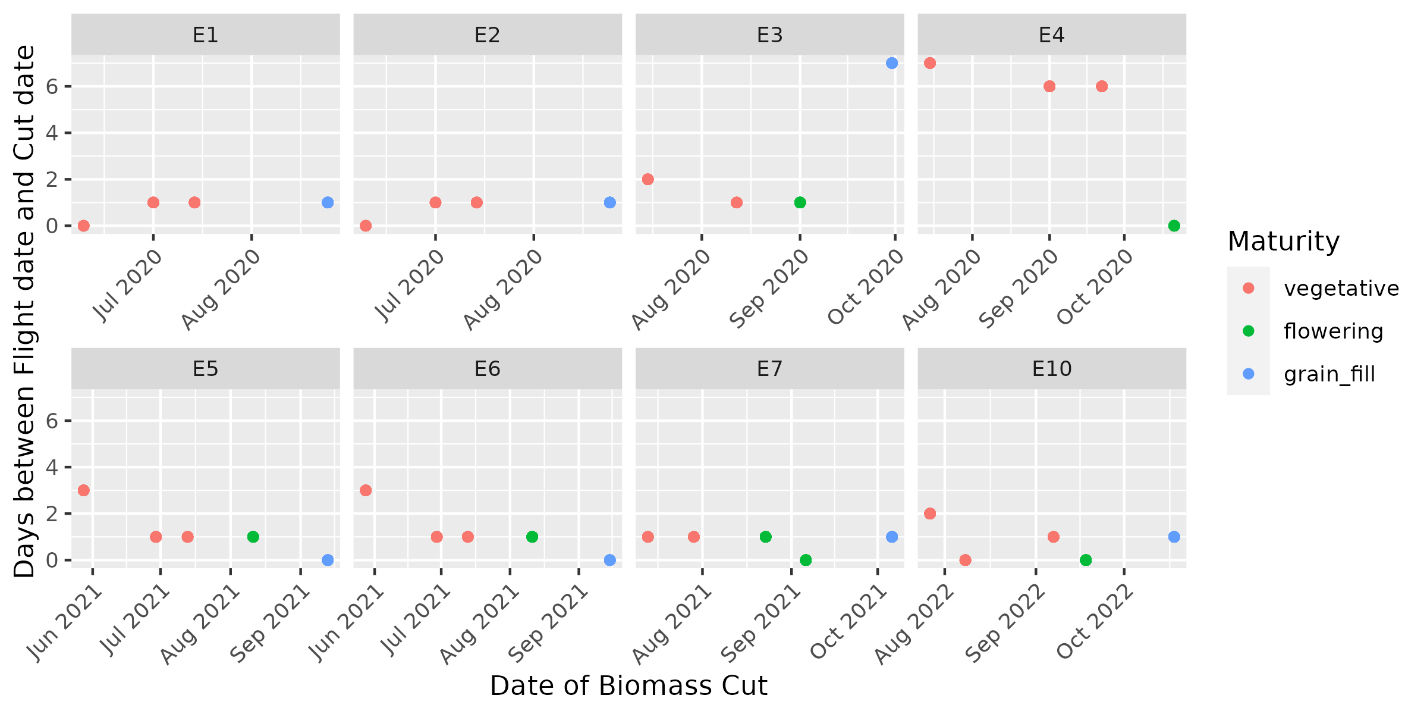


Fig. S 1 – Relationship between the Date of biomass cut and the date of the corresponding UAV flight for each experiment. Point colours represent the trial mean Zadok’s stage at a given timepoint.


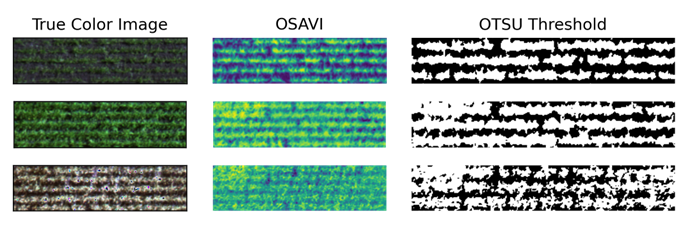


Fig. S 2 – Examples of the thresholding methodology that was used in the study. The panels include a true colour image, the OSAVI vegetation index and binary masks after thresholding using Otsu’s method.

| **Variable Set** | **Broad Growth-stage Group** | **Train set Observations** | **Test set Observations** | **Subset of Input Features** |
| --- | --- | --- | --- | --- |
| Combined | All | 1,270 | 314 | 24 |
| Combined | Vegetative | 715 | 186 | 24 |
| Combined | Flowering | 264 | 65 | 16 |
| Combined | Grain-Fill | 291 | 63 | 22 |
| Geometric | All | 1,270 | 314 | 6 |
| Geometric | Vegetative | 715 | 186 | 6 |
| Geometric | Flowering | 264 | 65 | 6 |
| Geometric | Grain-Fill | 291 | 63 | 7 |
| Vi | All | 1,270 | 314 | 20 |
| Vi | Vegetative | 715 | 186 | 19 |
| Vi | Flowering | 264 | 65 | 10 |
| Vi | Grain-Fill | 291 | 63 | 15 |

**Table S 1 –** Overview of number of samples included for each Variable and Growth-stage combination assessed in this study, including number of observations in the train set and test set, along with the number of input features included after filtering for co-linearity.

| **VarSet** | **Growth-stage** | **V1** | **V2** | **V3** | **V4** | **V5** | **V6** | **V7** | **V9** | **V10** | **V11** | **V12** | **V13** | **V14** | **V15** |
| --- | --- | --- | --- | --- | --- | --- | --- | --- | --- | --- | --- | --- | --- | --- | --- |
| Combined | All | volume height | AreaBelow50 height | AreaBelow75 height | AreaBelow25 height | stdev height | masked grvi | masked ng | masked rendvi | gari | masked varigreen | Coverage (%) osavi | masked endvi | nr | masked exg |
|  | Vegetative | volume height | AreaBelow25 height | clg | masked rvi | AreaBelow50 height | Coverage (%) osavi | endvi | gsavi | masked gndvi | masked ndvi | masked exg | masked vigreen |  |  |
|  | Flowering | volume height | AreaBelow75 height | masked rendvi | AreaBelow50 height |  |  |  |  |  |  |  |  |  |  |
|  | Grain_fill | r RedEdge | Coverage (%) osavi | gari | masked gari | Percentile 98 height |  |  |  |  |  |  |  |  |  |
| Geometric | All | volume height | AreaBelow50 height | AreaBelow75 height | AreaBelow25 height | stdev height |  |  |  |  |  |  |  |  |  |
|  | Vegetative | volume height | AreaBelow25 height | AreaBelow50 height | Coverage (%) osavi |  |  |  |  |  |  |  |  |  |  |
|  | Flowering | volume height |  |  |  |  |  |  |  |  |  |  |  |  |  |
|  | Grain_fill | Coverage (%) osavi | Percentile 98 height |  |  |  |  |  |  |  |  |  |  |  |  |
| Vi | All | ng | gemi | masked varigreen | rendvi | masked exg | masked grvi | masked clre | evi | masked rvi | exg | gari | masked endvi |  |  |
|  | Vegetative | clre | clg | ng | gsavi | masked ndvi | endvi | gemi | masked exg | exg | masked gsavi | masked vigreen | vigreen | masked osavi | masked gdvi |
|  | Flowering | masked rendvi | rvi | masked grvi |  |  |  |  |  |  |  |  |  |  |  |
|  | Grain_fill | r blue | r RedEdge | masked r green | gari | masked r RedEdge | rvi | masked evi | vigreen | msavi | masked varigreen | masked r nir |  |  |  |

Table S 2 - Overview of selected variables after recursive feature elimination (RFE) for each of the 12 variable x growth stage combinations. Text colour indicates whether a variable is geometric (blue) or spectral (orange). The numbering of the columns indicates the order in which each variable was selected.

|  |  |  | **Test** | | | | **Train** | | | |
| --- | --- | --- | --- | --- | --- | --- | --- | --- | --- | --- |
| **Growth-stage** | **Model** | **Variables** | **R2** | **rRMSE** | **RMSE** | **n** | **R2** | **rRMSE** | **RMSE** | **n** |
| all | PLSR_Refined | Combined | 0.88 | 0.28 | 164.45 | 314 | 0.89 | 0.26 | 159.36 | 1270 |
|  |  | Geometric | 0.81 | 0.35 | 202.12 | 314 | 0.81 | 0.34 | 207.55 | 1270 |
|  |  | Vi | 0.79 | 0.37 | 216.31 | 314 | 0.81 | 0.34 | 208.49 | 1270 |
|  | Random Forest | Combined | 0.99 | 0.08 | 46.39 | 314 | 0.99 | 0.08 | 46.6 | 1270 |
|  |  | Geometric | 0.93 | 0.22 | 127.87 | 314 | 0.93 | 0.2 | 125.29 | 1270 |
|  |  | Vi | 0.99 | 0.09 | 55.05 | 314 | 0.99 | 0.08 | 50.57 | 1270 |
|  | SVM | Combined | 0.91 | 0.25 | 143.2 | 314 | 0.92 | 0.22 | 133.54 | 1270 |
|  |  | Geometric | 0.82 | 0.35 | 202.06 | 314 | 0.81 | 0.34 | 211.45 | 1270 |
|  |  | Vi | 0.84 | 0.32 | 188.81 | 314 | 0.86 | 0.29 | 181.1 | 1270 |
|  | XGBoost | Combined | 1 | 0.05 | 31.2 | 314 | 1 | 0.05 | 29.47 | 1270 |
|  |  | Geometric | 0.93 | 0.22 | 129.94 | 314 | 0.92 | 0.22 | 138.85 | 1270 |
|  |  | Vi | 0.99 | 0.07 | 39.84 | 314 | 0.99 | 0.06 | 37.11 | 1270 |
| vegetative | PLSR_Refined | Combined | 0.85 | 0.26 | 68.76 | 186 | 0.89 | 0.23 | 64.51 | 715 |
|  |  | Geometric | 0.81 | 0.28 | 75.47 | 186 | 0.83 | 0.29 | 80.79 | 715 |
|  |  | Vi | 0.76 | 0.32 | 85.61 | 186 | 0.84 | 0.28 | 77.49 | 715 |
|  | Random Forest | Combined | 0.98 | 0.08 | 22.11 | 186 | 0.99 | 0.07 | 19.45 | 715 |
|  |  | Geometric | 0.92 | 0.18 | 48.99 | 186 | 0.93 | 0.19 | 53.08 | 715 |
|  |  | Vi | 0.98 | 0.1 | 25.79 | 186 | 0.99 | 0.08 | 22.59 | 715 |
|  | SVM | Combined | 0.87 | 0.24 | 62.81 | 186 | 0.91 | 0.21 | 58.55 | 715 |
|  |  | Geometric | 0.82 | 0.28 | 73.57 | 186 | 0.83 | 0.29 | 80.3 | 715 |
|  |  | Vi | 0.8 | 0.3 | 79.28 | 186 | 0.88 | 0.24 | 66.58 | 715 |
|  | XGBoost | Combined | 0.99 | 0.06 | 16.14 | 186 | 0.99 | 0.05 | 14.05 | 715 |
|  |  | Geometric | 0.93 | 0.18 | 46.95 | 186 | 0.94 | 0.18 | 51.06 | 715 |
|  |  | Vi | 0.99 | 0.08 | 20.79 | 186 | 0.99 | 0.07 | 18.27 | 715 |
| flowering | PLSR_Refined | Combined | 0.64 | 0.12 | 104.9 | 65 | 0.67 | 0.12 | 111.33 | 264 |
|  |  | Geometric | 0.55 | 0.13 | 116.79 | 65 | 0.58 | 0.14 | 124.87 | 264 |
|  |  | Vi | 0.34 | 0.16 | 143.66 | 65 | 0.45 | 0.16 | 144.3 | 264 |
|  | Random Forest | Combined | 0.89 | 0.07 | 58.78 | 65 | 0.88 | 0.08 | 67.95 | 264 |
|  |  | Geometric | 0.88 | 0.07 | 63.04 | 65 | 0.88 | 0.08 | 68.42 | 264 |
|  |  | Vi | 0.91 | 0.06 | 54.69 | 65 | 0.92 | 0.06 | 57.84 | 264 |
|  | SVM | Combined | 0.66 | 0.11 | 101.14 | 65 | 0.68 | 0.12 | 109.9 | 264 |
|  |  | Geometric | 0.55 | 0.13 | 116.35 | 65 | 0.59 | 0.14 | 124.52 | 264 |
|  |  | Vi | 0.36 | 0.16 | 139.69 | 65 | 0.45 | 0.16 | 145.43 | 264 |
|  | XGBoost | Combined | 0.88 | 0.08 | 67.74 | 65 | 0.9 | 0.08 | 72.11 | 264 |
|  |  | Geometric | 0.76 | 0.11 | 94.78 | 65 | 0.79 | 0.11 | 95.86 | 264 |
|  |  | Vi | 0.86 | 0.08 | 74.2 | 65 | 0.87 | 0.09 | 80.95 | 264 |
| grain_fill | PLSR_Refined | Combined | 0.84 | 0.15 | 180.22 | 63 | 0.83 | 0.14 | 171.02 | 291 |
|  |  | Geometric | 0.79 | 0.17 | 208.21 | 63 | 0.8 | 0.16 | 187.64 | 291 |
|  |  | Vi | 0.77 | 0.18 | 217.82 | 63 | 0.81 | 0.15 | 181.42 | 291 |
|  | Random Forest | Combined | 0.97 | 0.07 | 81.31 | 63 | 0.97 | 0.06 | 70.94 | 291 |
|  |  | Geometric | 0.95 | 0.09 | 110.72 | 63 | 0.96 | 0.07 | 87.26 | 291 |
|  |  | Vi | 0.97 | 0.07 | 85.77 | 63 | 0.97 | 0.06 | 70.12 | 291 |
|  | SVM | Combined | 0.85 | 0.15 | 178.89 | 63 | 0.83 | 0.14 | 169.03 | 291 |
|  |  | Geometric | 0.8 | 0.17 | 203.37 | 63 | 0.81 | 0.15 | 182.52 | 291 |
|  |  | Vi | 0.81 | 0.17 | 199.13 | 63 | 0.84 | 0.14 | 168.03 | 291 |
|  | XGBoost | Combined | 0.99 | 0.05 | 56.28 | 63 | 0.98 | 0.04 | 51.47 | 291 |
|  |  | Geometric | 0.92 | 0.12 | 143.85 | 63 | 0.93 | 0.1 | 117.89 | 291 |
|  |  | Vi | 0.99 | 0.04 | 52.63 | 63 | 0.99 | 0.04 | 44.72 | 291 |

Table S 3 – Train and Test performance metrics for each of the Growth-stage x variable set x Model types on the Permanent ROI.

| **Vegetation Index** | **Calculation** | **Name** | **Reference** |
| --- | --- | --- | --- |
| CLG | $\left( \frac{R_{NIR}}{R_{Green}} \right)-1$ | Grean Leaf Index | Gobron et al. (2000) |
| CLRE | $\left( \frac{R_{NIR}}{R_{RedEdge}} \right)-1$ | Chlorophyll Red Edge | Gitelson et al. (2005) |
| DVI | $R_{NIR}-R_{Red}$ | Difference Vegetation Index | Richardson and Wiegand (1977) |
| ENDVI | $\frac{\left( R_{NIR}+R_{Green} \right)-\left( 2*R_{Blue} \right)}{\left( R_{NIR}+R_{Green} \right)+\left( 2*R_{Blue} \right)}$ | Enhanced NDVI | MaxMax (2015) |
| EVI | $\frac{2.5*\left( R_{NIR}-R_{Red} \right)}{R_{NIR}+6*R_{Red}-7.5*R_{Blue}+1}$ | Enhanced Vegetation Index | Liu and Huete (1995) |
| EVI2 | $\frac{2.5*\left( R_{NIR}-R_{Red} \right)}{R_{NIR}+2.4*R_{Red}+1}$ | Enhanced Vegetation Index 2 | Jiang et al. (2008) |
| EXG | $2*R_{Green}-R_{Red}-R_{Blue}$ | Excess Green Index | Woebbecke et al. (1995) |
| GARI | $\frac{R_{NIR}-\left( R_{Green}-\left( R_{Blue}-R_{Red} \right) \right)}{R_{NIR}+\left( R_{Green}+\left( R_{Blue}+R_{Red} \right) \right)}$ | Green Atmospherically Resistant Vegetation Index | Gitelson et al. (1996) |
| GDVI | $R_{NIR}-R_{Green}$ | Green Difference Vegetation Index | Tucker (1979) |
| GEMI | $\frac{2*\left( {R_{NIR}}^{2}-{R_{Red}}^{2} \right)+1.5*R_{NIR}+0.5*R_{Red}}{\left( R_{NIR}+R_{Red}+0.5 \right)*(1-0.25* \frac{R_{Red}-0.125}{1-R_{Red}})}$ | Green Atmospherically Resistant Vegetation Index | Gitelson et al. (1996) |
| GLI | $\frac{2*R_{Green}-R_{Red}-R_{Blue}}{2*R_{Green}+R_{Red}+R_{Blue}}$ | Green Leaf Index | Gobron et al. (2000) |
| GNDVI | $\frac{R_{NIR}-R_{Green}}{R_{NIR}+R_{Green}}$ | Green Normalized Difference Vegetation Index | Gitelson et al. (1996) |
| GRVI | $\frac{R_{NIR}}{R_{Green}}$ | Green Ratio Vegetation Index | Gitelson et al. (2002) |
| GSAVI | $\frac{R_{NIR}-R_{Green}}{\left( R_{NIR}+R_{Green} \right)*1.5}$ | Green-Soil Adjusted Vegetation Index | Sripada (2005) |
| MSAVI | $\frac{2*R_{NIR}+{(\left( 2*R_{NIR}+1 \right)^{2}-8*\left( R_{NIR}-R_{Red} \right))}^{\frac{1}{2}}}{2}$ | Modified Soil-Adjusted Vegetation Index | Qi et al. (1994) |
| MSAVI2 | $\frac{2*R_{NIR}+1-\sqrt{\left( 2*R_{NIR}+1 \right)^{2}-8*\left( R_{NIR}-R_{Red} \right)}}{2}$ | Modified Soil-Adjusted Vegetation Index | Qi et al. (1994) |
| MTVI | $\frac{1.5*(1.2*\left( R_{NIR}-R_{Green} \right)-2.5*\left( R_{Red}-R_{Green} \right))}{\sqrt{\left( 2*R_{NIR}+1 \right)^{2}-\left( 6*R_{NIR}-5*\sqrt{R_{Red}} \right)-0.5}}$ | Modified Triangular Vegetation Index | Haboudane et al. (2004) |
| NDRE | $\frac{{R_{NIR}- R}_{RedEdge}}{R_{NIR}+R_{RedEdge}}$ | Normalized Difference Red Edge | Fitzgerald et al. (2006) |
| NDVI | $\frac{{R_{NIR}- R}_{Red}}{R_{NIR}+R_{Red}}$ | Normalized Difference Vegetation Index | Rouse et al. (1974) |
| NG | $\frac{R_{Green}}{R_{NIR}+R_{Green}+R_{Red}}$ | Normalized Green | Richards and Richards (2022) |
| NNIR | $\frac{R_{NIR}}{R_{NIR}+R_{Green}+R_{Red}}$ | Normalized NIR | Richards and Richards (2022) |
| NR | $\frac{R_{Red}}{R_{NIR}+R_{Green}+R_{Red}}$ | Normalized Red | Richards and Richards (2022) |
| OSAVI | $\frac{R_{NIR}-R_{Red}}{R_{NIR}+R_{Red}+0.16}*1.16$ | Optimized Soil-Adjusted Vegetation Index | Baret et al. (1993) |
| Blue | $R_{Blue}$ | Blue Reflectance | Richards and Richards (2022) |
| Green | $R_{Green}$ | Green Reflectance | Richards and Richards (2022) |
| Red | $R_{Red}$ | Red Reflectance | Richards and Richards (2022) |
| RedEdge | $R_{RedEdge}$ | RedEdge Reflectance | Richards and Richards (2022) |
| NIR | $R_{NIR}$ | NIR Reflectance | Richards and Richards (2022) |
| RVI | $\frac{R_{NIR}}{R_{Red}}$ | Ratio Vegetation Index | Jordan (1969) |
| SAVI | $\frac{R_{NIR}-R_{Red}}{R_{NIR}+R_{RED}+0.5}*1.5$ | Soil-Adjusted Vegetation Index | Huete (1988) |
| VARIGreen | $\frac{R_{Green}-R_{Red}}{R_{Green}+R_{Red}-R_{Blue}}$ | Visible Atmospherically Resistand Index Green | Gitelson et al. (2001) |
| VIGreen | $\frac{R_{Green}-R_{Red}}{R_{Green}+R_{Red}}$ | Vegetation Index Green | Zarco-Tejada et al. (2001) |

Table S 4 – Abbreviation, calculation, name, and key reference for the Spectral indices used as input to biomass prediction models in this study.

| Geometric Trait | Calculation | Citation |
| --- | --- | --- |
| Coverage % OSAVI | Vegetation Pixels determined by Otsu thresholding of vegetation index: 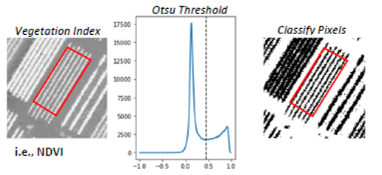 i.e OSAVI  Let $n$ be the number of pixels in the region of interest:  $Num Vegetation Pixels= \sum_{i=1}^{n} \left\{ \begin{aligned} 1 if vegetation \\ 0 otherwise \end{aligned} \right.$  $Coverage \%=\frac{Num Vegetation Pixels}{n}*100$ | Marcial-Pablo et al. (2019); Otsu (1979) |
| Percentile_height | Let $H$ be the set of all height values in the region of interest. Calculate the percentile height $P$ for a desired percentile (i.e 75^th^ Percentile):  Sort the heights: $h_{1}\leq h_{2}\leq\ldots\leq h_{n}$  Find the index: $k=\left[ 0.75*n \right]$  Percentile height: $P=H_{k}$ | Wang et al. (2022) |
| Area Below Height | Count the number of pixels below the percentile height $P$:  $Num Pixels below P= \sum_{i=1}^{n} \left\{ \begin{aligned} 1 if h_{i}<P \\ 0 otherwise \end{aligned} \right.$  $Total Area below P=Num Pixels below P*GSD$ | Das et al. (2022) |
| Canopy Volume | $Sum of Heights= \sum_{i=1}^{n} h_{i}$  Where $h_{i}$represents the height of the ith pixel.  $Canopy Volume=\frac{Sum of Heights*GSD}{Area of ROI}$ | Maimaitijiang et al. (2019) |
| Standard Deviation Height | $Standard Deviation= \sqrt{\frac{1}{n}\sum_{i=1}^{n} \left( h_{i}- \bar{h} \right)^{2}}$  Where $h_{i}$ represents the height of the $i$th pixel and $\bar{h}$ is the mean height | Das et al. (2022) |

Table S 5 - Calculation of Geometric Variables for use as input variables for biomass prediction models. Table includes the name of the trait, a description of how it was calculated, and a relevant reference.


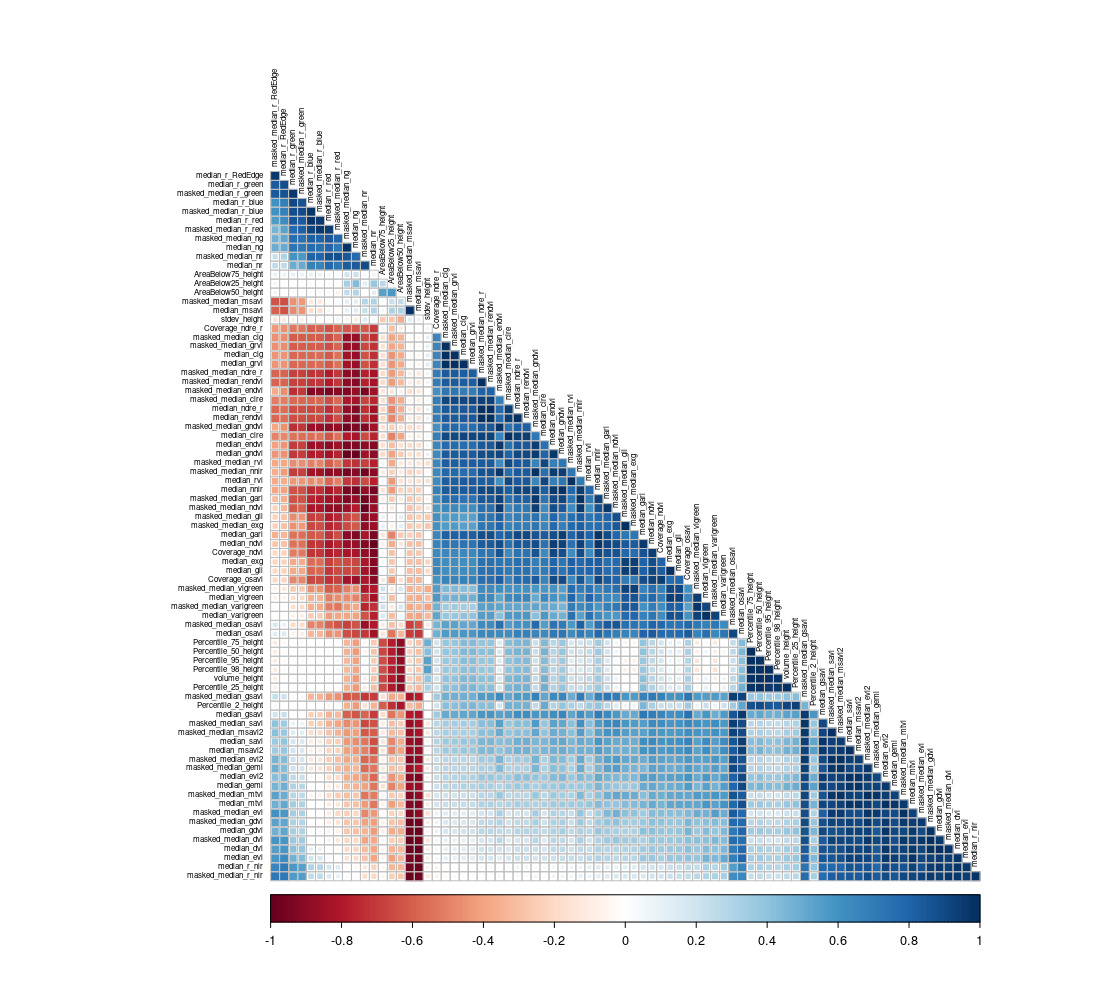


Fig. S 3 – Correlation Matrix illustrating the relationship between all variables and all timepoints, ordered by the angle of eigenvectors (AOE).


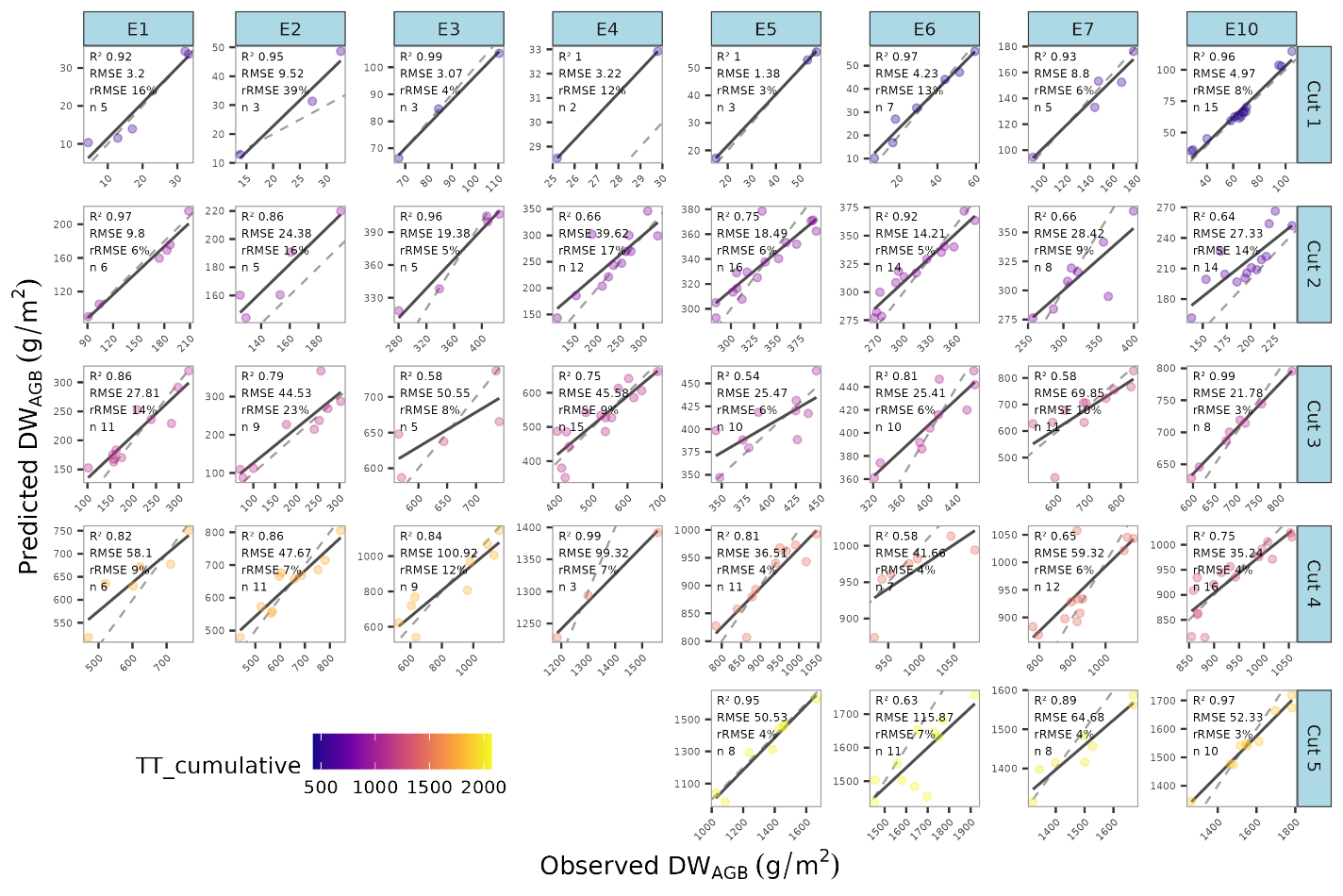


Fig. S 4 - Observed versus predicted DW_AGB_ (g/m^2^) using the Random Forest model trained using geometric and spectral variables on the independent test set. Vertical facets represent the different experiments in the study and the horizontal facets represent the DW_AGB_ cuts in numeric order. Point colours represent the cumulative thermal time (TT_cumulative_). Metrics for each cut x experiment are shown in each facet.
